# Angiogenic gene signature in human pancreatic cancer correlates with TGF-beta and inflammatory transcriptomes

**DOI:** 10.18632/oncotarget.6345

**Published:** 2015-11-18

**Authors:** Kelly E. Craven, Jesse Gore, Julie L. Wilson, Murray Korc

**Affiliations:** ^1^ Departments of Biochemistry and Molecular Biology, Indiana University School of Medicine, Indianapolis, IN 46202, USA; ^2^ Department of Medicine, Indiana University School of Medicine, Indianapolis, IN 46202, USA; ^3^ The Pancreatic Cancer Signature Center at Indiana University Simon Cancer Center, Indianapolis, IN 46202, USA

**Keywords:** pancreatic cancer, TCGA, angiogenesis, TGF-β, inflammation

## Abstract

Pancreatic ductal adenocarcinomas (PDACs) are hypovascular, but overexpress pro-angiogenic factors and exhibit regions of microvasculature. Using RNA-seq data from The Cancer Genome Atlas (TCGA), we previously reported that ∼12% of PDACs have an angiogenesis gene signature with increased expression of multiple pro-angiogenic genes. By analyzing the recently expanded TCGA dataset, we now report that this signature is present in ∼35% of PDACs but that it is mostly distinct from an angiogenesis signature present in pancreatic neuroendocrine tumors (PNETs). These PDACs exhibit a transcriptome that reflects active TGF-β signaling, and up-regulation of several pro-inflammatory genes, and many members of JAK signaling pathways. Moreover, expression of SMAD4 and HDAC9 correlates with endothelial cell abundance in PDAC tissues. Concomitantly targeting the TGF-β type I receptor (TβRI) kinase with SB505124 and JAK1-2 with ruxolitinib suppresses JAK1 phosphorylation and blocks proliferative cross-talk between human pancreatic cancer cells (PCCs) and human endothelial cells (ECs), and these anti-proliferative effects were mimicked by JAK1 silencing in ECs. By contrast, either inhibitor alone does not suppress their enhanced proliferation in 3D co-cultures. These findings suggest that targeting both TGF-β and JAK1 signaling could be explored therapeutically in the 35% of PDAC patients whose cancers exhibit an angiogenesis gene signature.

## INTRODUCTION

Pancreatic ductal adenocarcinoma (PDAC) is the fourth leading cause of cancer-related deaths in the United States, with a 5-year survival rate of 7% [1]. Several aspects of PDAC pathobiology contribute to this poor prognosis. First, approximately 80% of PDAC patients are diagnosed at an advanced stage with locally invasive and/or metastatic disease which precludes the option of life-prolonging tumor resection [2]. Second, PDAC is associated with several high frequency driver mutations, a plethora of low frequency driver mutations, and excessive production of growth factors and their receptors, leading to the activation of multiple aberrant signaling pathways that promote chemoresistance [3–5]. Third, PDAC exhibits intense tumor desmoplasia and a complex tumor microenvironment (TME) that is rich in collagens, hyaluronan, and fibronectin [6–9], harbors inflammatory cells and macrophages, and is generally hypovascular and hypoxic [6–9]. Consequently, there is compression of the existing vasculature, and attenuated drug delivery into the pancreatic tumor mass [6–8, 10]. Fourth, there are few gene signatures or biomarkers that will allow for the rational design of targeted therapies to specific subgroups of PDAC patients. One such example is the use of PARP inhibitors in patients with genomic instability and either BRCA-1 or -2 mutations, or PALB-2 mutations [4, 11, 12].

Based on analysis of preliminary PDAC transcriptome data from The Cancer Genome Atlas (TCGA), we previously reported that ∼12% of PDACs exhibit a pro-angiogenic gene signature [13]. In the present study, we analyzed the recently expanded TCGA dataset which includes more PDAC cases to understand the relationship between this signature and the presence of intratumoral endothelial cells. The expanded dataset also includes pancreatic neuroendocrine tumors (PNETs), and in contrast to the findings in PDAC, PNETs are often highly vascular, yet the prognosis of these patients is better than that of patients with PDAC, even in metastatic PNET [14]. Although PNETs account for approximately 2% of all pancreatic tumors [15], understanding the differences between PNETs and PDAC in relation to angiogenesis could provide a better understanding for the failure of anti-angiogenic therapy in PDAC, and could shed new light on the reasons for the vastly better prognosis of PNET in spite its propensity to metastasize to the liver.

We now report that a strong angiogenesis gene signature is present in ∼35% of PDAC cases, and is mostly distinct from the angiogenic genes up-regulated in PNETs. The same PDAC cases also exhibit a strong TGF-β signaling signature. Moreover, in a custom-prepared tissue microarray (TMA) of PDAC tissues, strong SMAD4 immunoreactivity in the cancer cells correlates with enhanced microvessel density (MVD). PDACs in TCGA, but not PNETs, are also enriched in genes implicated in inflammation and JAK/STAT signaling. Our previous findings demonstrated that ruxolitinib alone suppressed mitogenic effects by murine endothelial cells on co-cultured murine pancreatic cancer cells (PCCs). By contrast, we now show that concomitant inhibition of TGF-β signaling with SB505124 and JAK signaling with either ruxolitinib or JAK1-targeting shRNA is required to impede human PCC and human endothelial cell (EC) mitogenic cross-talk in 3D co-culture of both cell types. Therefore, we propose that this combination could represent a novel therapeutic approach in PDAC patients whose cancers exhibit an angiogenesis gene signature and SMAD4-positive cancer cells.

## RESULTS

### PDACs and PNETs have distinct angiogenesis gene signatures

**Figure 1:**
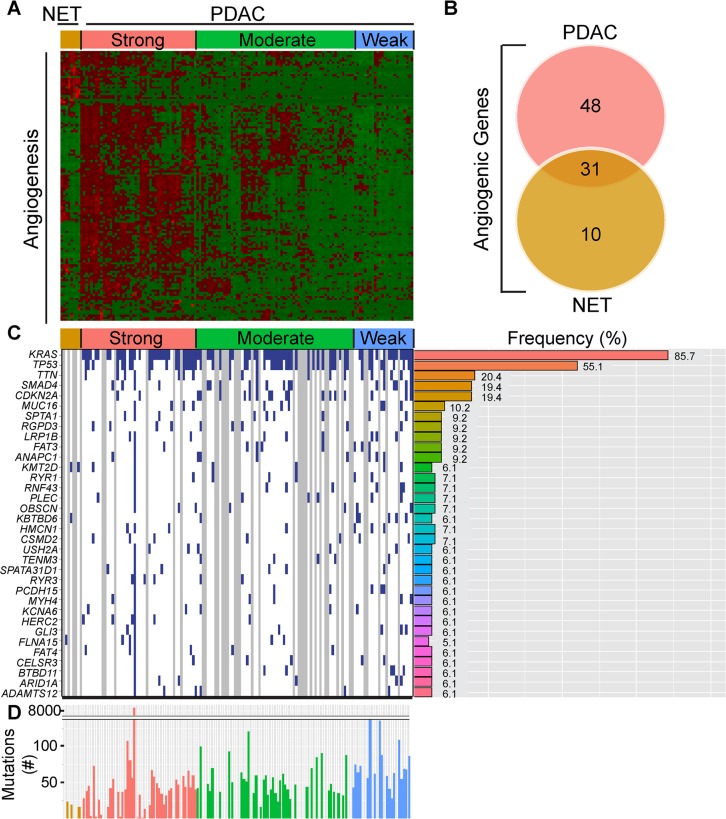
PDACs have varying degrees of an angiogenic gene signature that is distinct from PNETs (**A**) Hierarchical clustering of RNA-Seq expression values from 129 angiogenic genes in 8 PNET (NET) and 135 PDAC TCGA samples confirms the presence of PDAC subgroups with strong, moderate, and weak expression of these genes (red = up-regulated; green = down-regulated). (**B**) Differential expression analysis of the 129 genes between the strong angiogenic PDAC group vs. the weak angiogenic PDAC group and the NET group vs. weak angiogenic PDAC group reveals that 31 angiogenic genes are common to both tumor types, whereas 48 and 10 are unique to strong angiogenic PDACs or PNETs, respectively (Fold Change > = 1.5, False Discovery Rate (FDR) < 0.05). (**C**) The top 34 non-silently mutated genes that appear with a frequency of 6% or more in the 3 NET + 98 PDAC cases combined are listed on the left while their mutation frequencies in PDAC are graphed to the right. Samples (left) appear in the same order as the cluster analysis with blue indicating a non-silently mutated gene, white a silently mutated or wild type gene, and gray denotes samples that lack mutation data. (**D**) The total number of non-silently mutated genes for NET and PDAC samples was graphed. Median counts are as follows: NET: 19, PDAC: 47, Strong Angio PDAC: 40, Moderate Angio PDAC: 48, Weak Angio PDAC: 64.

In contrast to the well-vascularized pancreatic neuroendocrine tumors (PNETs), pancreatic ductal adenocarcinomas (PDACs) are dense and relatively hypovascular. Nevertheless, PDACs harbor endothelial cells and some exhibit regions rich in microvasculature [13]. To assess angiogenic gene expression in PDAC, we analyzed pancreatic tumor RNA-Seq data from The Cancer Genome Atlas (TCGA). This TCGA dataset was recently expanded to 178 patient samples that include PDAC and PNET cases. Given the known vascular nature of PNETs, their vastly better prognosis by comparison to PDACs, the known propensity of both tumor types to metastasize to the liver, and the association of tumor angiogenesis with the metastatic process, we also examined angiogenic gene expression in PNETs. We focused our analysis on PNETs (*n* = 8) and PDACs that lacked secondary or unknown histopathological characteristics (*n* = 135), and assessed the expression levels of 129 angiogenesis genes that we identified by cluster analysis of PDAC RNA-Seq data [13]. Hierarchical clustering revealed that ∼35% of PDACs (47/135) grouped together and exhibited up-regulation of multiple angiogenesis genes, whereas ∼47% (64/135) and ∼18% (24/135) had increased expression of some or few of these genes (Figure 1A). Thus, there are three subgroups of PDAC, each with distinct angiogenesis gene expression profiles that we termed as having strong, moderate or weak angiogenic gene signatures. By contrast, all 8 PNETs grouped together and exhibited increased expression of a subset of angiogenesis genes (Figure 1A).

To identify genes up-regulated in PDACs with a strong signature and to assess overlap with genes up-regulated in PNETs, we next conducted a differential expression analysis comparing the strong PDAC subgroup or PNETs with the weak subgroup. Out of 129 angiogenesis genes, 79 were significantly up-regulated in PDACs with a strong signature whereas 41 were up-regulated in PNETs (Supplementary Table 1). Comparison of these gene lists revealed that 31 genes were up-regulated in both PDACs and PNETs, including *FGFR1*, and *VEGFR-1* (*FLT1*), −*2* (*KDR*) and −*3* (*FLT4*) all of which are pro-angiogenic (Figure 1B, Supplementary Table 1). By contrast, 48 genes were significantly up-regulated only in PDAC (Figure 1B, Table 1), and 22 of these genes were directly connected (Supplementary Figure 1). Conversely, 10 genes were unique to PNETs, and only 2 were functionally connected but in an indirect manner (Figure 1B, Table 2, Supplementary Figure 1). Thus, many angiogenesis genes are up-regulated in PNETs and a subgroup of PDACs, and while the expression of some genes overlaps between these tumor types, PDAC exhibits increased expression of a larger set of functionally connected angiogenesis genes.

**Table 1:** Genes unique to the PDAC angiogenesis gene signature

Number	Gene Symbol	Fold Change	*P*-value	FDR
1	*ACVRL1*	1.76	1.04E-04	7.61E-04
2	*ANGPT1*	3.38	4.31E-08	8.07E-07
3	*APOD*	2.07	2.25E-03	1.04E-02
4	*C3*	2.1	1.30E-05	1.24E-04
5	*C3AR1*	3.23	2.08E-11	1.04E-09
6	*C6*	2.32	5.79E-03	2.28E-02
7	*CCR2*	5.7	3.24E-13	2.90E-11
8	*CLIC4*	2.75	5.92E-12	3.47E-10
9	*CMA1*	11.66	1.09E-03	5.65E-03
10	*COL15A1*	3.2	2.23E-15	4.01E-13
11	*COL4A3*	3.72	1.81E-03	8.67E-03
12	*CXCL12*	3.48	2.35E-11	1.16E-09
13	*CYP1B1*	7.4	1.51E-29	1.50E-25
14	*ECSCR*	1.69	4.01E-03	1.69E-02
15	*ELK3*	2.62	1.16E-08	2.57E-07
16	*ENPEP*	2.73	2.38E-08	4.86E-07
17	*EPAS1*	1.65	2.06E-04	1.37E-03
18	*GJA5*	1.87	1.07E-04	7.77E-04
19	*GNA13*	1.73	2.31E-04	1.50E-03
20	*GPR124*	2.89	1.72E-12	1.21E-10
21	*GREM1*	3.82	2.90E-04	1.83E-03
22	*HAND2*	2.28	1.47E-04	1.02E-03
23	*HDAC9*	1.82	9.78E-03	3.50E-02
24	*HIF1A*	2.01	2.14E-06	2.54E-05
25	*HIPK1*	1.63	5.16E-04	3.00E-03
26	*ITGAV*	2.2	1.10E-07	1.86E-06
27	*ITGB1*	1.68	3.43E-04	2.11E-03
28	*JAM3*	1.75	2.41E-04	1.56E-03
29	*MAP3K7*	1.57	4.48E-03	1.84E-02
30	*MEOX2*	3.63	2.35E-08	4.81E-07
31	*NRP1*	2.38	8.17E-10	2.59E-08
32	*PIK3CA*	2.24	1.89E-06	2.27E-05
33	*PIK3CG*	5.16	1.46E-12	1.06E-10
34	*PLXDC1*	1.95	3.71E-05	3.11E-04
35	*PLXND1*	1.63	1.40E-03	6.98E-03
36	*PTEN*	1.57	7.89E-04	4.29E-03
37	*ROBO1*	3.82	1.68E-16	4.23E-14
38	*ROCK1*	1.67	1.49E-03	7.35E-03
39	*ROCK2*	1.74	3.24E-04	2.02E-03
40	*SIRT1*	1.67	1.56E-03	7.65E-03
41	*STAB1*	2.29	2.32E-08	4.76E-07
42	*TEK*	3.74	1.13E-14	1.57E-12
43	*TGFBR1*	1.53	9.04E-03	3.28E-02
44	*TGFBR2*	1.86	4.36E-05	3.56E-04
45	*THBS4*	5.55	7.95E-11	3.27E-09
46	*THSD7A*	4.72	7.58E-11	3.14E-09
47	*TIE1*	2.21	1.46E-07	2.35E-06
48	*WASF2*	1.58	1.28E-03	6.46E-03

**Table 2:** Genes unique to the PNET angiogenesis gene signature

Number	Gene Symbol	Fold Change	*P*-value	FDR
Up-regulated				
1	*ANGPTL3*	17.34	6.42E-03	2.41E-02
2	*BAI3*	47.69	2.18E-06	2.31E-05
3	*FGF9*	3.84	6.96E-03	2.56E-02
4	*GTF2I*	2.22	9.34E-05	6.47E-04
5	*HIPK2*	2.66	1.52E-06	1.67E-05
6	*ISL1*	19.37	1.82E-06	1.97E-05
7	*SCG2*	65.37	7.42E-06	6.87E-05
8	*SRPK2*	2.14	6.70E-04	3.59E-03
9	*TSPAN12*	3.37	2.04E-04	1.27E-03
10	*VEZF1*	1.73	7.18E-03	2.63E-02

### PDAC subgroups have similar mutation profiles

To determine whether the angiogenesis gene signature present in 35% of PDACs is associated with a specific mutational burden, we gleaned curated mutation data from TCGA using version 1.2.0 which included information from 98 PDAC and 3 PNET cases. Overall, PDACs exhibited more mutations (median = 47) than PNETs (median = 19) (Figure 1C), raising the possibility that differences in mutational burden could account for divergent angiogenic profiles. However, mutations were similar across the PDAC subgroups, and consistent with PDAC genome sequencing studies [3, 4, 16, 17], *KRAS* (∼86%), *TP53* (∼55%), *SMAD4* (19%) and *CDKN2A* (19%) were four of the five most frequently mutated genes (Figure 1C). Given that the anticipated mutation frequencies of *SMAD4* and *CDKN2A* are 50% and 90%, respectively [18], these observations suggest that TCGA may underestimate the frequency of certain driver mutations. We therefore analyzed copy number data to determine whether either of these tumor suppressor genes are deleted. *SMAD4* and *CDKN2A* deletions were present in ∼14% and ∼26% of PDACs, respectively (Supplementary Figure 2A), indicating that *SMAD4* and *CDKN2A* inactivation arises from both mutations and homozygous deletions. We next assessed whether any genes have different mutational frequencies across the PDAC subgroups. From > 9800 mutated genes, only *KBTBD6* which has no known role in angiogenesis, was differentially mutated when comparing the strong and weak subgroups (*P* < 0.05; Supplementary Table 2). No other genes were differentially mutated, and the mean number of mutated genes in each PDAC patient was similar. Thus, specific gene mutations and overall mutational burden do not necessarily explain the different angiogenic signatures in PDAC.

### PDAC vessel density correlates with the presence of SMAD4

**Figure 2:**
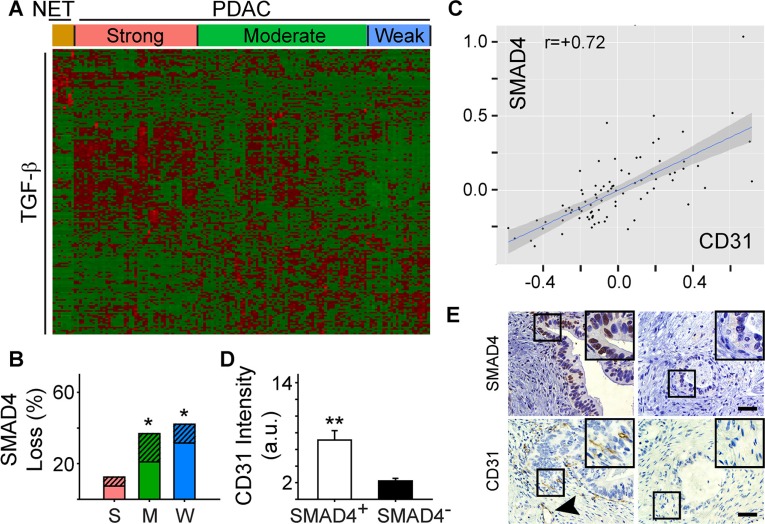
Angiogenic gene signatures correlate with increased expression of TGF-β target genes (**A**) While preserving the order of the 8 PNET (NET) and 135 PDAC TCGA patient samples according to the angiogenesis cluster analysis, hierarchical clustering of RNA-Seq expression values from 186 TGF-β responsive genes indicated that a subset of TGF-β target genes are up-regulated in the strong angiogenic PDAC group (red = up-regulated; green = down-regulated). (**B**) Overall *SMAD4* loss by mutation or deletion was significantly higher in the moderate (M) and weak (W) angiogenic PDAC subgroups compared with the strong subgroup (S) as determined using TCGA. Stacked bars show the total % of patients in each subgroup with *SMAD4* mutations (solid bars) or deletions (hatched bars). *P < 0.05. (**C**) Analysis of protein expression data from TCGA shows that SMAD4 and CD31 levels correlate in PDAC. (**D**) Quantification of CD31 and SMAD4 immunostaining of a human PDAC tissue microarray (TMA) shows that in SMAD4-positive tumors (open bar), CD31 immunoreactivity is significantly higher than in SMAD4-negative tumors (closed bar). **P < 0.01. (**E**) Representative images of the SMAD4 and CD31 immunostaining on the TMA shows that CD31-positive endothelial cells and vessels (arrowhead) are present in PDACs with SMAD4 immunoreactivity in the PCCs (left panels), whereas in SMAD4-negative tumors CD31 immunoreactivity is rarely present (right panels). Insets show magnified images of boxed areas. Scale bars 50 μm.

We next sought to determine whether specific pathway alterations could explain the different angiogenic gene signatures present in PDAC. Accordingly, we subjected the 79 differentially expressed angiogenesis genes to Ingenuity Pathway Analysis (IPA). IPA identified TGF-β as a significant upstream regulator of their expression (*P* = 1.17 ×10^−11^) suggesting that PDACs with a strong angiogenic signature could also exhibit a TGF-β gene signature. To explore this possibility, we performed hierarchical clustering which preserved the order of patient samples that clustered together in the angiogenesis analysis, but was focused on a dataset of 186 TGF-β target genes from the gene set enrichment analysis (GSEA) Molecular Signatures Database (MSigDB). In the strong PDAC subgroup, a subset of TGF-β target genes were up-regulated and were distinct from targets up-regulated in PDACs with moderate or weak angiogenesis signatures (Figure 2A). Overall, 50 TGF-β target genes were increased when comparing the strong and weak PDAC subgroups, including pro-angiogenic *CTGF* and *ITGA5* (Supplementary Table 3). Moreover, *ITGB1*, *NRP1* and *FLT4*(Supplementary Table 1), were increased in PDACs with a strong angiogenic signature, and all of these are TGF-β targets (Supplementary Table 3). Thus, PDACs with a strong angiogenic signature exhibit increased expression of many TGF-β target genes.

Notably, TGF-β3 was significantly increased in the strong PDAC subgroup (fold increase: 3.4; *P*-value: 1.89 × 10^−12^; FDR 1.31 × 10^−10^), whereas TGF-β1 and TGF-β2 were not differentially expressed. Moreover, *SMAD4* inactivation by mutation or deletion only occurred in ∼13% of cases in the strong subgroup, but ∼37% and ∼42% of cases in the moderate and weak subgroups, respectively (Figure 2B, Supplementary Figure 2B). Thus, we analyzed protein array data from the PDAC TCGA dataset to investigate the relationship between SMAD4 expression and the levels of the endothelial cell-specific marker, CD31 (Cluster of Differentiation 31). PDACs with high levels of SMAD4 expressed high levels of CD31, whereas low levels of SMAD4 were associated with low CD31 (Figure 2C). To confirm these observations, we assessed SMAD4 and CD31 protein expression in a tissue microarray (TMA) of 54 human PDAC tissues using CD31- and wild-type SMAD4-detecting antibodies [19]. Nuclear SMAD4 was present in the cancer cell nuclei of 23 PDACs in which CD31-positive endothelial cells (ECs) and vessels were abundant (Figure 2D–2E). By contrast, wild-type SMAD4 immunoreactivity was not detectable in the cancer cells in 31 PDACs, and in these tissues CD31 immunoreactivity was sparse (Figure 2D–2E). These data therefore suggest that the presence of wild-type SMAD4 in pancreatic cancer cells correlates with endothelial cell abundance.

### PDAC vessel density also correlates with the presence of HDAC9

**Figure 3:**
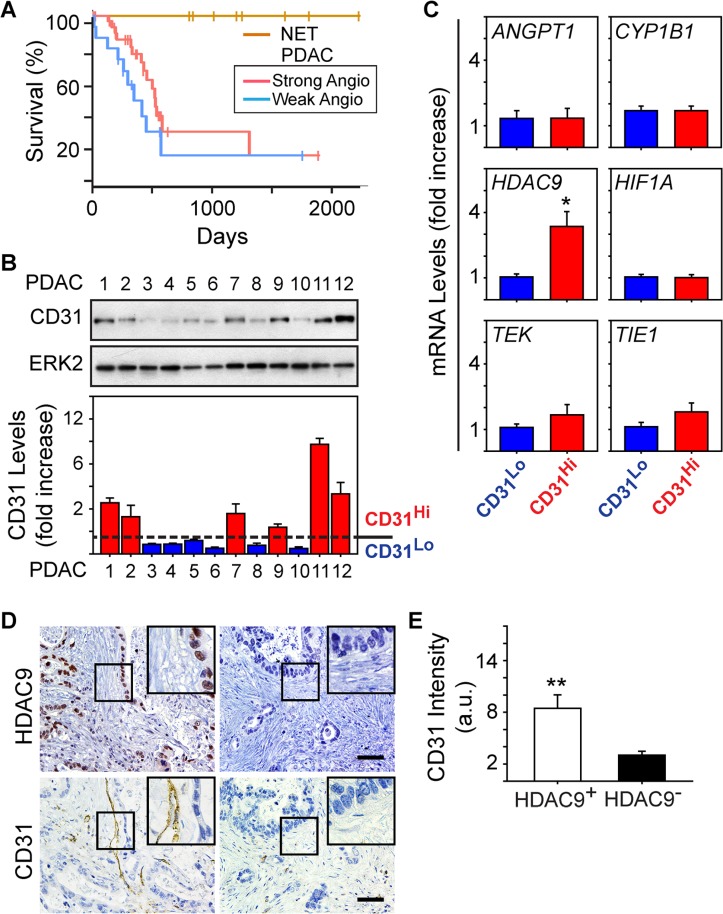
HDAC9 correlates with microvessel density in PDAC tumors (**A**) Kaplan-Meier survival plot of patients presenting with Stage IIB PDAC and whose tumors express a strong (*n* = 34, median: 592 days) or weak (*n* = 15, median: 393 days) angiogenic profile shows no significant difference in overall survival (*P*-value: 0.17) between the two groups, whereas PNET (NET) survival is significantly longer than both PDAC subgroups (*P* < 0.01). (**B**) Immunoblots with human PDAC tissue homogenates show that CD31 is present in some PDACs, but is nearly undetectable in others. ERK2 confirms equivalent lane loading. Quantification of three independent immunoblots confirms that 6 PDACs exhibited relatively high levels of CD31 (CD31^Hi^; red bars) whereas the other 6 PDACs had lower levels (CD31^Lo^; blue bars). (**C**) Quantitative PCR for the indicated mRNAs shows that compared with CD31^Lo^ PDACs (blue bars), CD31^Hi^ PDACs (red bars) express higher levels of HDAC9 whereas the other mRNAs were not different between these groups. *P < 0.05 (**D**) Immunohistochemistry shows that PDACs with strong nuclear HDAC9 immunoreactivity in the PCCs harbor CD31-positive endothelial cells and vessels (left panels), whereas in PDACs in which HDAC9 is undetectable, CD31 immunoreactivity is rarely present (right panels). Insets show magnified images of boxed areas. Scale bars 50 μm. (**E**) Quantification of CD31 immunostaining shows that in HDAC9-positive tumors (open bar), CD31 immunoreactivity is significantly higher than in HDAC9-negative tumors (closed bar). **P < 0.01.

We next assessed patient survival to determine whether the strong angiogenesis gene signature could be useful for PDAC prognosis. The majority of PDAC cases in the TCGA dataset are Stage IIB (73%). Therefore, we extracted survival information for these patients to compare overall survival between patients whose tumors are similar with respect to staging, but different with respect to their angiogenesis signatures. Kaplan-Meier analysis revealed that the median survival of patients in the strong angiogenesis subgroup was 592 days, and in the weak subgroup it was 393 days (Figure 3A). By contrast, there were no observed PNET deaths, with a median follow-up of 1410 days, and a log rank analysis comparing all three groups confirmed that PNET survival was significantly longer than the survival of PDAC patients (*P* < 0.01; Figure 3A). However, there were no statistically significant differences in survival between the PDAC subgroups (*P* = 0.17; Figure 3A). Thus, an angiogenesis signature is not necessarily prognostic in PDAC, but there is a tendency for patients with a strong angiogenesis signature to survive longer than patients who lack this signature.

Given these observations, we next sought to determine whether the strong angiogenesis gene signature could be useful for predicting vessel abundance (termed microvessel density: MVD) in PDAC. Accordingly, we analyzed resected PDAC tissues for CD31 as a marker of endothelial cell abundance and overall MVD, and angiogenic genes up-regulated in the strong subgroup. From the 12 PDACs analyzed, 50% exhibited moderate to high levels of CD31 (termed CD31^Hi^) whereas in the other 50%, CD31 was low to nearly undetectable (termed CD31^Lo^) (Figure 3B). To determine whether CD31^Hi^ tumors also express high levels of angiogenic genes, we next compared *ANGPT1*, *TIE1*, *TEK*, *CYP1B1*, *HIF1A* and *HDAC9* levels in CD31^Hi^ tumors with their levels in CD31^Lo^ tumors. We selected these genes because all six were present in the angiogenesis signature unique to PDAC (Table 1), because *CYP1B1* was the most significantly and highly up-regulated, and because *ANGPT1*, its receptors *TIE1* and *TEK*, and *HIF1A* are commonly associated with pathways that enhance tumor angiogenesis [20, 21]. *HDAC9* is also pro-angiogenic [22], and we recently reported that it is required for murine pancreatic cancer cells derived from the KRC (oncogenic Kras combined with loss of RB) genetically engineered mouse model to stimulate proliferation of SV40-transformed murine endothelial cells (SVEC4-10; [13]). Whereas *ANGPT1*, *TIE1*, *TEK*, *CYP1B1* and *HIF1A* mRNA levels were similar when comparing CD31^Hi^ with CD31^Lo^ PDACs, HDAC9 levels were markedly up-regulated in CD31^Hi^ tumors (Figure 3C). Moreover, 34/54 (∼63%) PDACs in the TMA exhibited strong HDAC9 immunoreactivity in the cancer cells, and in these tumors, CD31-positive endothelial cells were abundant (Figure 3D–3E). By contrast, CD31-positive endothelial cells were rarely detected in PDACs without HDAC9 immunoreactivity (20/54) (Figure 3D–3E). Therefore, HDAC9 is a member of the angiogenesis gene signature that correlates with endothelial cell abundance and microvessel density in PDAC.

### PDACs with an angiogenesis gene signature are enriched in inflammation-related genes

**Figure 4:**
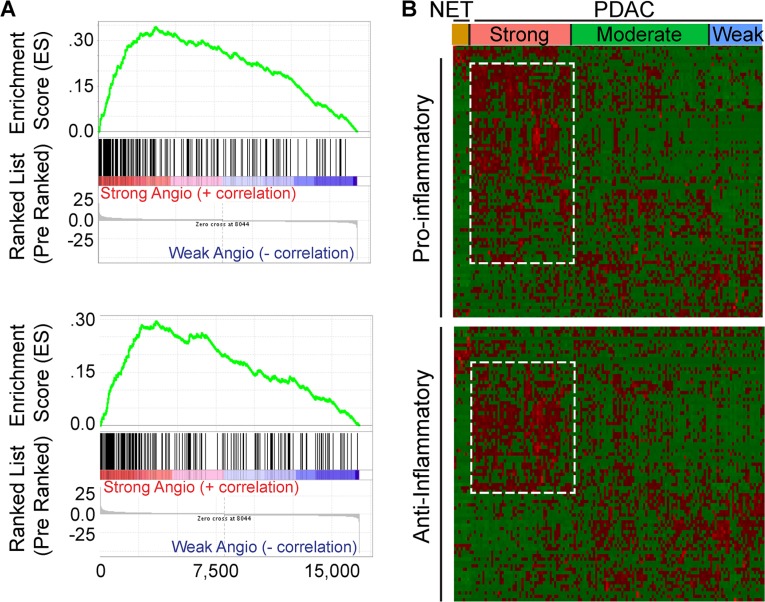
Angiogenic PDACs have an inflammatory profile (**A**) Gene Set Enrichment Analysis (GSEA) indicates that genes up-regulated during transplant rejection (top) or during the inflammatory response (bottom) correlate with genes up-regulated in the strong angiogenic PDAC subgroup when compared to the weak angiogenic PDAC subgroup (family-wise error rate (FWER) < 0.001). (**B**) While preserving the order of the 8 PNET (NET) and 135 PDAC TCGA patient samples according to the angiogenesis cluster analysis, hierarchical clustering of RNA-Seq expression values from 85 positive (top) or 81 negative (bottom) regulators of inflammation indicates that the strong angiogenic PDAC subgroup up-regulates subsets (outlined) of both positive and negative regulators of inflammation (red = up-regulated; green = down-regulated). Differential expression analysis reveals up-regulation of 28 positive and 16 negative regulators of inflammation and down-regulation of 7 positive and 17 negative regulators of inflammation, suggesting a tipping of the scale to a pro-inflammatory environment (|Fold Change > = 1.5|, False Discovery Rate (FDR) < 0.05).

STAT3 is an oncogene and survival factor that can exert pro-angiogenic effects downstream of multiple inflammatory factors [23, 24]. We recently reported that active STAT3 is often present in cancer, stromal and endothelial cells in human PDAC tissues, and in tumors arising in the KRC mouse model [13]. Within murine endothelial cells, STAT3 is activated by pancreatic cancer cell-derived pro-inflammatory and pro-angiogenic factors, leading to the up-regulation of HDAC9 which then enhances murine endothelial cell proliferation [13]. It is not known, however, whether these pathways are associated with angiogenesis in human PDAC. Therefore, we conducted a GSEA comparing differentially expressed genes between the strong and weak PDAC subgroups with gene sets related to inflammation. This analysis revealed that the transcriptome of PDACs with a strong angiogenesis gene signature correlated strongly with gene expression profiles arising from inflammatory responses (Figure 4A). Moreover, leading edge analysis of these GSEAs indicated that genes up-regulated in the strong PDAC subgroup were enriched in pro-inflammatory genes, including *IL1B*, *IL6* and *JAK2*, anti-inflammatory *IL10*, as well as *HDAC9*. Next, hierarchical clustering was carried out to assess the levels of all genes annotated as either positive (+) or negative (−) regulators of inflammation in each of the PDAC subgroups. PDACs with a strong angiogenesis signature exhibited increased expression of multiple genes annotated to (+) inflammation, and a subset of (−) inflammation genes (Figure 4B). However, differential expression analysis comparing the strong and weak angiogenesis subgroups revealed that only 16 (−) inflammation genes were up-regulated, whereas 17 were down-regulated (Supplementary Table 4). By contrast, 28 (+) inflammation genes were up-regulated (Supplementary Table 5), indicating that PDACs with a strong angiogenesis signature are associated with increased expression of several transcripts that have the capacity to promote inflammation.

### TβRI and JAK inhibition blocks angiocrine effects

**Figure 5:**
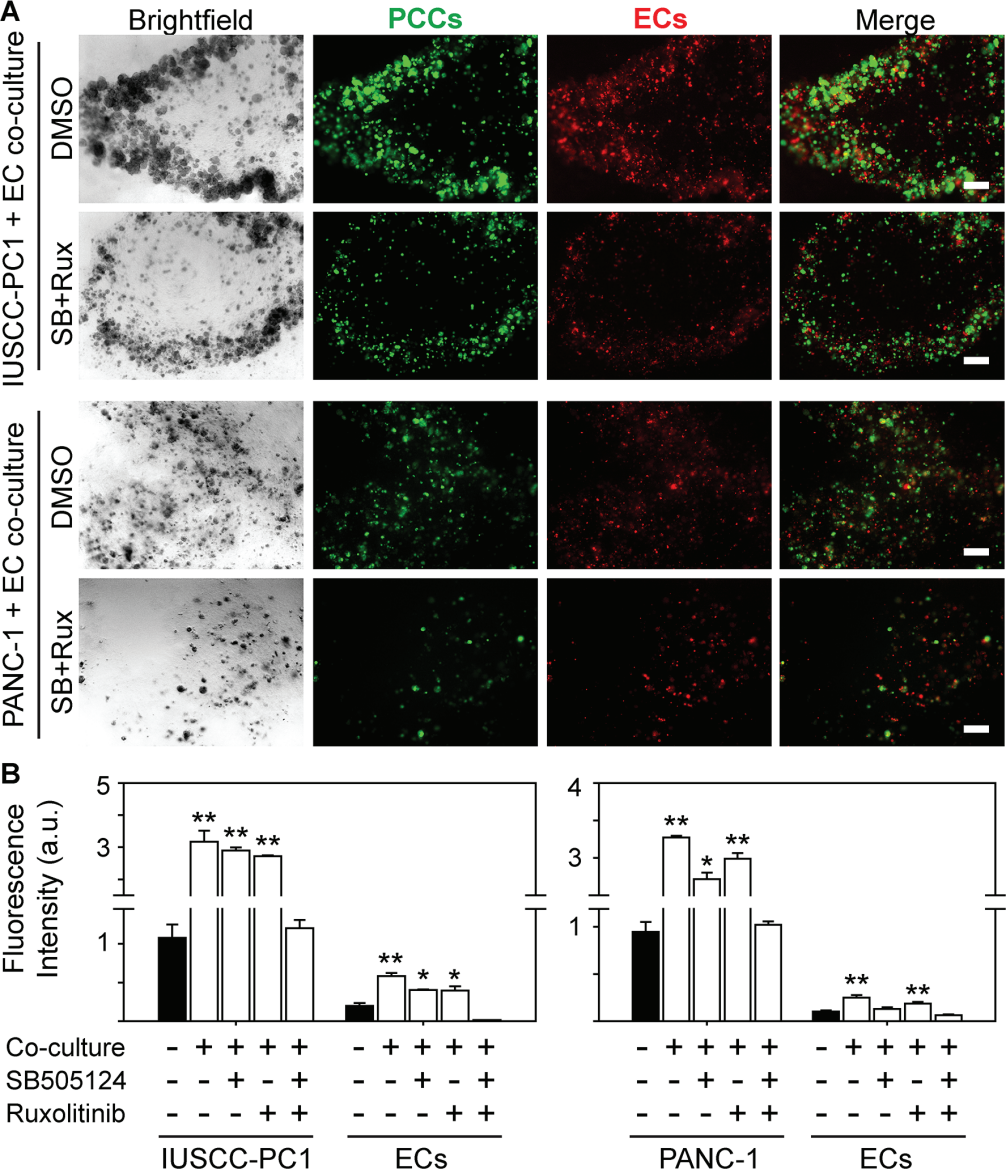
TβRI and JAK1-2 inhibition suppress human PCC and EC growth (**A**) 3D co-cultures of IUSCC-PC1 or PANC-1 human PCCs (green) and human ECs (HUVECs, red) shows that compared with vehicle (DMSO [0.05%]), ruxolitinib [100 nM] together with SB505124 [2 μm] suppress PCC and EC growth. Shown are representative brightfield and fluorescent images from three independent experiments. Scale bars, 200 μm. (**B**) Fluorescence intensity quantification shows that compared with 3D cultures in which IUSCC-PC1 or PANC-1 PCCs and HUVECs are cultured independently (closed bars), culturing ECs and PCCs together in 3D (open bars) significantly enhances PCC and EC growth, which is completely blocked when ruxolitinib and SB505124 are combined, but not by either inhibitor alone. Data are mean ± SEM from three independent experiments. *P < 0.05, and **P < 0.01.

Our findings indicate that PDACs in the strong angiogenesis subgroup were associated with increased expression of TGF-β target genes and an inflammation signature in which several pro-inflammatory genes, including Janus kinase 2 (*JAK2*) were increased. Moreover, the strong angiogenesis subgroup expressed wild-type SMAD4 protein that correlated with CD31 levels. Therefore, we next sought to determine whether inhibition of TGF-β type I receptor (TβRI) and/or JAK signaling pathways can block angiogenic effects. We used the well-known PANC-1 human pancreatic cancer cells (PCCs), as well as our recently established IUSCC-PC1 cell line in 3D co-cultures with non-immortalized human vascular endothelial cells (HUVECs). Both PANC-1 cells and IUSCC-PC-1 cells harbor mutated *KRAS* (*KRAS^G12D^*) and express wild-type *SMAD4* as determined by DNA sequencing. Importantly, establishing new PCCs diminishes the potential for additional mutations and clonal selections that may arise from serial *in vivo* and *in vitro* passaging. Thus, IUSCC-PC-1 cells were used to confirm findings with PANC-1 cells which were established in 1975 [25]. To monitor changes in the growth for each cell type in the co-culture studies, PCCs and HUVECs were labeled with green and red fluorescence, respectively. By comparison to 3D cultures in which PCCs and HUVECs were cultured separately, IUSCC-PC-1 and PANC-1 cell proliferation was significantly enhanced by co-culture with HUVECs (Figure 5A–5B). Moreover, HUVEC proliferation was enhanced in co-cultures with either PCC line (Figure 5A–5B). These mitogenic effects were completely blocked by the combination of the JAK inhibitor, ruxolitinib, and the TβRI inhibitor, SB505124, but not by either inhibitor alone (Figure 5A–5B). These results therefore suggest that human PCCs exert growth-promoting angiogenic effects on endothelial cells, that endothelial cells exert mitogenic effects on PCCs, and that these events are suppressible by combinatorial targeting of JAK and TGF-β signaling pathways.

### JAK1 mediates endothelial cell growth

**Figure 6:**
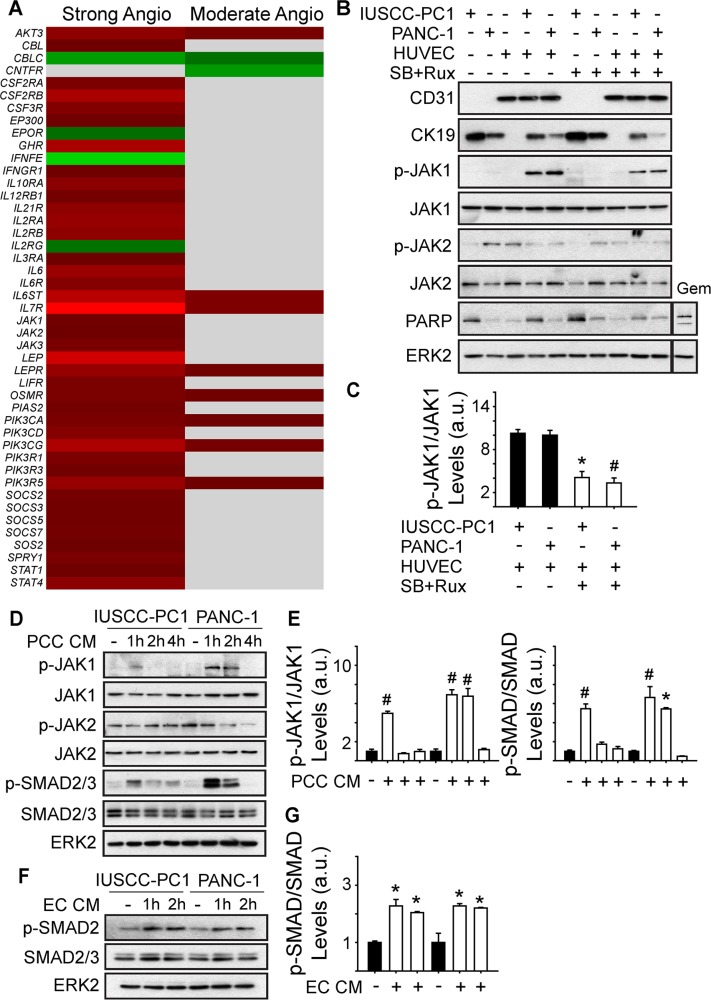
Angiogenic PDACs are enriched in JAK-STAT signaling genes (**A**) Compared with the weak angiogenic PDAC subgroup, 40 or 8 JAK/STAT signaling pathway genes are up-regulated in the Strong or Moderate Angiogenic groups, respectively. (**B**) Immunoblots with 3D culture lysates from IUSCC-PC1 or PANC-1 human PCCs with or without HUVECs (ECs) show that endothelial (CD31) and epithelial (CK19) markers are present in PCC:EC co-cultures, in which p-JAK1 but not p-JAK2 levels are markedly increased. SB505124 [2 μm] together with ruxolitinib [100 nM] suppresses p-JAK1, but does not induce PARP cleavage. By contrast, gemcitabine (Gem, [10 μM]) enhances cleaved PARP levels in co-cultured cells. (**C**) Quantification confirms that SB505124 and ruxolitinib (open bars) significantly decreases p-JAK1 levels in co-cultured cells. (**D–G**) Immunoblots with EC (D) or PCC (F) lysates show that conditioned media (CM) from PCCs increases p-JAK1 and p-SMAD levels in ECs, whereas CM from ECs increases p-SMAD levels in PCCs. Quantification confirms that PCC CM (E, open bars) significantly increases p-JAK1 and p-SMAD in ECs, and that EC CM (G, open bars) significantly increases p-SMAD in PCCs. ERK2 in B, D and F confirms equivalent lane loading. Data in C, E and F are presented as mean ± SEM from three independent experiments. *P < 0.05, ^#^*P* < 0.01.

**Figure 7:**
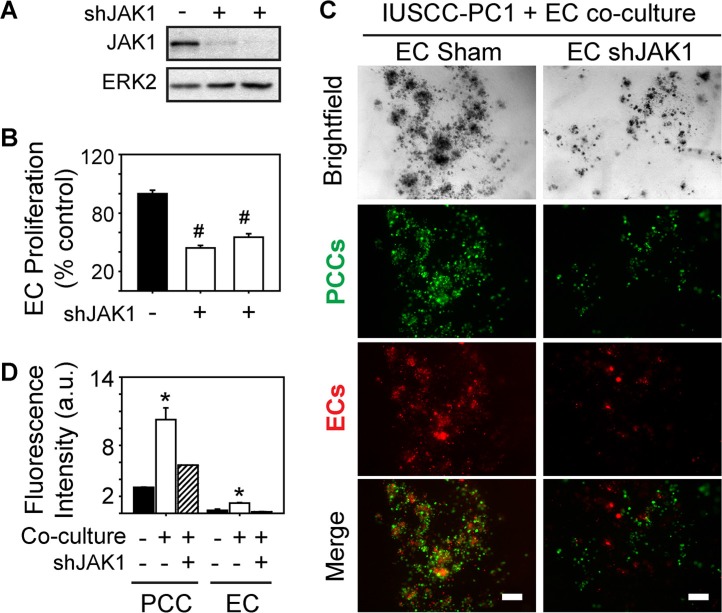
JAK1 is required for endothelial cell growth and angiocrine effects on pancreatic cancer cells (**A–B**) Two different JAK1-targeting shRNAs decrease JAK1 expression in HUVECs (A), and significantly decrease HUVEC proliferation (B, open bars) compared with Sham-transduced control cells. Cell proliferation was assessed using MTT assay, and data are presented as mean ± SEM from three independent experiments. ERK2 in (A) confirms equivalent lane loading. (**C**) 3D co-cultures with IUSCC-PC1 PCCs (green) and HUVECs (red) transduced with a non-targeting control (EC Sham) or JAK1 shRNA (EC shJAK1) shows that JAK1 knockdown in ECs suppresses PCC and EC growth. Shown are representative brightfield and fluorescent images from three independent experiments. Scale bars, 200 μm. (**D**) Fluorescence intensity quantification confirms that JAK1 knockdown in ECs suppresses PCC and EC growth in 3D co-cultures (hatched bars). Data are mean ± SEM from three independent experiments. *P < 0.05, ^#^*P* < 0.01.

Analysis of genes involved in JAK signaling revealed that 40 genes in this pathway were up-regulated in PDACs with a strong angiogenic signature, including *JAK1*, *JAK2* and *JAK3* (Figure 6A, Supplementary Table 6). Because ruxolitinib selectively targets JAK1 and JAK2 [26], we next assessed whether one or both of these kinases are involved in the mitogenic cross-talk between human PCCs and HUVECs. Phosphorylated JAK1 (p-JAK1) was only detectable in co-cultures of PCCs and HUVECs, and its levels were suppressed by the combination of SB505124 and ruxolitinib (Figure 6B–6C). By contrast, JAK2 phosphorylation was not affected by co-culture, or by SB505124 and ruxolitinib (Figure 6B). Moreover, this combination failed to induce cleaved PARP in PCCs or HUVECs, or in co-cultures of these cells (Figure 6B). To determine whether increased p-JAK1 occurred in HUVECs, PCCs or both cell types, we added conditioned media from IUSCC-PC1 or PANC-1 cells to HUVECs, and, conversely, conditioned media from HUVECs to PCCs. Conditioned media from the PCCs markedly enhanced p-JAK1 levels and induced SMAD phosphorylation in HUVECs (Figure 6D–6E). By contrast, conditioned media from HUVECs failed to induce JAK1 phosphorylation in the PCCs (not shown), but stimulated SMAD phosphorylation (Figure 6F–6G). Thus, JAK1 activation is endothelial cell-specific whereas canonical TGF-β signaling pathways are activated in both cell types. To assess the role of JAK1 in endothelial cell growth, and in mediating angiocrine effects on PCCs we used shRNAs to suppress JAK1 expression in HUVECs. Both shRNAs markedly attenuated JAK1 expression levels in HUVECs, and suppressed their proliferation and ability to stimulate PCC growth (Figure 7A–7D). Therefore, endothelial JAK1 is required for the angiocrine effects of endothelial cells on PCCs.

## DISCUSSION

PDACs are desmoplastic and hypoxic tumors. Nonetheless, PDACs exhibit foci of EC proliferation, and a positive correlation has been reported between blood vessel density, tumor VEGF-A levels, and increased frequency of hepatic metastases and disease progression in PDAC [27–29]. Despite these observations, PDACs are refractory to VEGF-A-targeted therapies [30–32]. However, recent clinical trials have raised the possibility that targeting several pro-angiogenic signaling pathways may be beneficial in PDAC. For example, the VEGF and PDGF receptor inhibitor vatalanib was recently shown to slightly improve survival in metastatic PDAC [33], and TL-118, which combines four agents with a potential to inhibit angiogenesis, has also demonstrated promising results in early phase clinical trials [34]. Taken together, these observations underscore the need to have an improved understanding of the role of angiogenesis in PDAC.

In the present study, we utilized RNA-seq data from the recently updated pancreatic tumor TCGA dataset to explore relationships between angiogenesis and angiogenic gene expression levels. In agreement with our previous findings [13], this transcriptome analysis served to divide PDACs into three distinct subgroups based on their overall angiogenic gene expression profiles. Importantly, the recently expanded TCGA data for PDAC revealed that the strong angiogenesis signature is present in ∼35% of PDACs, underscoring the prevalence of this gene profile in PDAC. The expanded dataset also afforded the opportunity to conduct a preliminary analysis of angiogenic gene expression in PNETs. All 8 of the PNET cases currently available for analysis exhibited a similar angiogenic profile, but some of these genes were distinct from those up-regulated in PDAC. For example, PNETs exhibited marked increases in *ANGPTL3*, *ISL1* and *SCG2* expression. Angiopoietin-like 3 (*ANGPTL3*) stimulates endothelial cell migration and vessel formation [35], whereas Islet-1 (*ISL1*) and secretoneurin which is processed from *SCG2*, enhance endothelial growth and survival [36–38] and both of these genes are commonly associated with PNETs [39–42]. By contrast, other genes overlapped with PDACs, including *BMPER*, *GPLD1*, and *NXRN1* and *NXRN3*. *BMPER* is pro-angiogenic, and enhances endothelial migration and tube formation by stimulating FGF receptor (FGFR) signaling [43, 44]. Although targeting FGFR signaling is effective in murine PDAC and PNET models [45, 46], *FGFR-2* was markedly decreased in PNETs. FGFR-2 exists as two major splice isoforms with different C-terminal portions in the Ig-like region closest to the intracellular domain, and both isoforms have been implicated in PDAC biological aggressiveness [47, 48]. Therefore, the marked decrease in FGFR-2 expression in PNETs, which has not been previously reported, may contribute to the attenuated aggressiveness of PNETs by comparison with PDAC. By contrast, *GPLD1*, *NXRN1* and *NXRN3* were markedly increased in PNETs. Phospholipase D1 (encoded for by *PLD1* genes) and neurexins (encoded for by *NXRN* genes) are also pro-angiogenic, and exert their effects by modulating VEGF and TIE-2 receptor signaling [49–53]. Given that the fold increases in expression for these angiogenic genes were greater in PNETs than in PDACs, these observations raise the possibility that they could contribute to the grossly angiogenic phenotype of PNETs.

PDACs exhibited increased expression of many more angiogenic genes that included a larger set of pro-angiogenic genes distinct from those up-regulated in PNETs. For example, *ANGPT1*, *COL15A1*, *COL4A3*, *CXCL12* (SDF-1), and *ITGAV* and *ITGB1* were only up-regulated in PDACs, and together constituted part of a complex network of functionally connected genes. ANGPT1 enhances endothelial cell survival through the receptor tyrosine kinase, TIE2 [20], whereas SDF-1, collagens and integrins enhance endothelial cell migration and adhesion [54–56]. Together, these observations underscore the complexity of angiogenesis in PDAC, and the divergence between PDAC and PNET in relation to their angiogenic gene expression profiles. Moreover, these findings highlight the presence of multiple pro-angiogenic pathways in PDAC, which may explain why PDACs evade therapies that target single angiogenic pathways [30, 32] while PNETs respond to bevacizumab and to the angio-kinase inhibitors sunitinib and sorafenib [57–59].

The TGF-β type I (TβRI) and type II receptors (TβRII) were also up-regulated in PDACs but not in PNETs, and investigation into pathways through which the strong angiogenic PDAC signature could arise revealed that patients who have this signature also have signatures rich in TGF-β targets and inflammation-related genes. All three TGF-β isoforms are overexpressed in PDAC and are associated with poor outcome [60], and within the tumor microenvironment TGF-βs modulate angiogenesis, but enhance or suppress it depending on which genes are regulated and how they influence the angiogenic switch [9, 61]. For example, restoration of canonical TGF-β signaling in SMAD4-deficient pancreatic cancer cells (PCCs) has been reported to suppress angiogenesis by up-regulating anti-angiogenic thrombospondin-1 (*THBS1*) [62], and we detected a 5.63-fold increase in *THBS1* in patients with a strong angiogenic signature. However, SMAD4 re-expression in BxPC3 PCCs fails to suppress angiogenesis *in vivo* [63], and blocking TGF-β signaling suppresses tumor growth, metastasis, and angiogenesis in orthotopic mouse models [13, 64, 65]. Moreover, disrupting canonical TGF-β signaling by SMAD4 deletion suppresses metastasis in a genetically engineered mouse model (GEMM) of PDAC [66]. We recently reported that canonical TGF-β pathways stimulate pro-angiogenic gene expression in murine PCCs [13], and in the present study we determined that SMAD4 correlates with endothelial cell abundance in human PDAC tissues, suggesting that active canonical TGF-β signaling pathways promote angiogenesis in PDAC.

Inflammatory cells from different lineages enhance tumor angiogenesis by secreting pro-inflammatory and pro-angiogenic cytokines into the tumor milieu [9, 67]. Here, we identified inflammatory genes that were only elevated within the strong angiogenic PDAC subgroup that included multiple members of JAK-STAT signaling pathways, and both positive and negative regulators of inflammation. Overall, 16 negative regulators of inflammation were increased, and several of these are pro-angiogenic. For example, *ADIPOQ* which rescues impaired angiogenesis in ADIPOQ-null mice [68, 69] and stimulates endothelial migration and tube formation [70] was increased by ∼11-fold, whereas pro-angiogenic *GHSR* and *NLRP3* [71–74] were increased by ∼4.8- and ∼4-fold, respectively. Moreover, up-regulated positive regulators of inflammation included *TLR2* and *TLR7*, both of which are pro-angiogenic [75], and IL6, the IL-6 receptor (*IL6ST*) and all three Janus kinases (*JAK1-3*). *TLR7* activates STAT3 and markedly accelerates pancreatic tumorigenesis in GEMMs [76], whereas the IL-6 receptor (*IL6ST*) forms a signaling cascade with JAKs which activates STAT3 to enhance endothelial growth [77]. STAT3 is an important pro-survival factor in PDAC [78] and enables PCCs from the KRC GEMM to stimulate murine EC growth by up-regulating HDAC9 [13]. HDAC9 is a class IIa histone deacetylase that mediates pro-inflammatory cytokine release from macrophages [79, 80], polarizes naïve T-cells into regulatory *T*-cells [81, 82], and exerts angiogenic effects in endothelial cells [13, 22]. In the present study, we determined that HDAC9 is strongly expressed in the cancer cells in PDAC tissues with abundant vasculature, raising the possibility that HDAC9 could be a new marker for angiogenesis in PDAC.

In summary, our study highlights marked congruity between pro-inflammatory genes, TGF-β target genes, and the presence of a strong angiogenic signature. Thus, targeting inflammatory and/or TGF-β pathways in PDAC patients could serve to disrupt tumor-promoting angiogenic effects. In support of this conclusion, targeting these pathways together using SB505124 and ruxolitinib suppressed endothelial JAK1 activation in 3D co-cultures of human pancreatic cancer cells (PCCs) and human endothelial cells (ECs), and prevented ECs from exerting proliferative angiocrine effects on PCCs. This observation is consistent with our previous findings using a murine model system, but demonstrates that such a mechanism also exists in human ECs and PCCs, and that these effects are mediated in part, by endothelial JAK1. Taken together, the findings in the current study suggest that targeting TGF-β together with JAK1 pathways could be an advantageous approach to suppress cancer cell growth and angiogenesis in PDAC, and raise the possibility that this combination may be especially effective in patients whose tumors have wild-type SMAD4, exhibit HDAC9-positivity, or express an angiogenic gene signature.

## MATERIALS AND METHODS

### Hierarchical clustering, differential expression and gene set enrichment analysis

Normalized RNA-Seq RSEM [83] reads and raw count reads were downloaded from the TCGA pancreatic ductal adenocarcinoma dataset (PAAD) on December 31, 2014 from http://cancergenome.nih.gov/. This transcriptome dataset includes 183 patient samples. 179 of these are tumor samples (2 from one patient), and 4 are matched normal. Thus, there are 178 different tumor samples. Of these, 135 are confirmed PDAC and 8 are PNET. Other tumors (14/178) have additional histopathological characteristics, whereas 21/178 are mucinous colloid carcinomas, undifferentiated carcinomas or an unknown histological type. Hierarchical clustering was performed in R using data from PNETs (*n* = 8) or confirmed PDACs without additional histopathological characteristics (*n* = 135). Normalized RSEM values of 129 angiogenesis genes [13] were centered and scaled, and rows were clustered using a Pearson correlation distance and average linkage function, and columns were clustered using a Euclidean distance and complete linkage function. The centered and scaled expression values were graphed as a heatmap (red = up-regulated; green = down-regulated) in combination with the associated row and column dendrograms using the heatmap.2 function of the gplots R package.

For TGF-β, 192 HUGO gene names annotated to the “TGFB_UP.V1_UP” gene set were obtained from the gene set enrichment analysis (GSEA) Molecular Signatures Database. 190 of these had associated HGNC IDs, which were mapped to 190 Entrez gene IDs using the *Homo sapiens* gene file from NCBI. Four genes were excluded because 2 were already part of the angiogenic signature and 2 did not have TCGA RNA-Seq information. Clustering of 186 genes was performed as described above, but columns were not clustered. Instead, they were arranged in the same order as in the angiogenesis profile. Differential expression analyses were performed using DESeq [84] on the raw count data. Statistical criteria for differential gene expression were (|Fold Change (FC)| ≥ 1.5; FDR < 0.05).

For inflammation, GSEAs were performed using the Hallmark Gene Sets collection in the GSEA Molecular Signatures Database and the GSEAPreranked tool (v.2.2.0) on a ranked list of genes that were sorted according to fold change differences between the strong and weak PDAC subgroups [85, 86]. Clustering and differential expression was performed as described above using UniProt IDs annotated to positive regulation of inflammation (GO:0050729) or negative regulation of inflammation (GO:0050728) and the BioMart Bioconductor package to convert ids to Entrez gene ids [87–90].

To assess angiogenesis gene interactions, we used GeneMANIA [91]. Briefly, the angiogenesis genes unique to PNET (10) or PDAC (48), or genes that were common to both (31) were analyzed using GeneMANIA's pathway datasets. Any genes that were not directly connected were removed from the resulting network. For JAK-STAT analysis, 145 genes in the KEGG_JAK_STAT_SIGNALING_PATHWAY pathway were obtained from GSEA. Fold changes and FDRs for these genes were isolated from the DESeq analysis, and differential expression was defined as (|Fold Change| > = 1.5; FDR < 0.05), and fold changes were graphed in a heatmap using the heatmap.2 function of the gplots R package. Red or green represent up- or down-regulation, respectively. Gray represents genes not differentially expressed.

### Mutation, deletion and protein expression analysis

The curated mutation maf file (v.1.2.0) was downloaded on December 31, 2014 and included information from 98 PDAC cases and 3 PNET cases. Silent mutations were removed. Then, a matrix of genes and samples was built with each gene being coded as 1 (non-silently mutated), 0 (silently mutated or not mutated) or NA (no data available). Therefore, multiple non-silent mutations in a gene would only be counted once per gene. R was used to plot the mutation profiles of the samples in the same order as they appear in the cluster analysis. Significant differences in mutational frequencies were determined using a Fisher's exact test. *P* < 0.05 was considered statistically significant.

PDAC TCGA copy number data (level 4) was downloaded from the April 2, 2015 Broad GDAC Firehose GISTIC analysis run [92, 93]. The all_thresholded.by_genes.txt file was used, which classifies genes as having a copy number of −2 (deep loss, possibly a homozygous deletion), −1 (shallow loss, possibly a heterozygous deletion), 0 (diploid), 1 (low-level amplification), or 2 (high-level amplification). Of the PNET and PDAC samples, all 8 PNETs and 134 PDACs had copy number data. R was used to plot copy number profiles in the same order in the cluster analyses. For combining *SMAD4* mutation and deletion frequency, we used 97 PDAC samples that had both mutation and copy number data, with 40, 38, and 19 samples belonging to the Strong, Moderate, and Weak Angiogenic groups, respectively. Frequencies of samples with a mutation or deep deletion in SMAD4 were compared among the groups using a Fisher's exact test, with *P* < 0.05 considered significant.

Normalized reverse phase protein array (RPPA) values were downloaded from the TCGA PDAC dataset. Of the 135 PDAC used in the hierarchical clustering, 85 PDAC samples had protein expression information. CD31 and SMAD4 protein expression values for the 85 PDAC samples were used for graphing and correlation calculation.

### Survival analysis

Survival information for PDAC and PNET patients was downloaded on December 31, 2014 from http://cancergenome.nih.gov/. All patients had associated clinical information on days to death or days to last follow-up (censored). Overall survival was plotted using a Kaplan-Meier curve.

### Cell lines and 3-dimensional (3D) culture

PANC-1 (CRL-1469) pancreatic cancer cells (PCCs) were from ATCC. The IUSCC-PC-1 cell line was established from a patient-derived orthotopic xenograft in an athymic mouse [13]. IUSCC-PC-1 cells were authenticated, and confirmed to be human and free of pathogens and other cell types by IDEXX Bioresearch (St. Louis, MO). By sequencing, the cell line harbored a *KRAS* mutation (*KRAS^G12D^*) but lacked *SMAD4* mutations, and readily formed tumors in nude mice. HUVEC (CRL-1730) endothelial cells (ECs) were from ATCC. PCCs were cultured in DMEM with 1% antibiotic (100 units/ml penicillin; 100 mg/ml streptomycin) and 5% FBS. HUVECs were cultured in EGM-2 medium (Lonza, Walkersville, MD). HUVECs were transduced with lentivirus containing JAK1-targeting shRNAs or a non-targeting control shRNA from Thermo Fisher (Waltham, MA) as described [94]. PCCs and HUVECs were fluorescently-labeled before culturing in 3D as described [13].

### Immunohistochemistry

The paraffin-embedded human PDAC tissue microarray (TMA) was obtained from the Tissue Procurement and Distribution core at the Indiana University Simon Cancer Center, and 4 μm sections were prepared. Immunohistochemistry was performed as described [65] using SMAD4 (Leica Biosystems, Buffalo Grove, IL), HDAC9 (Origene, Rockville, MD) or CD31 (BD Biosciences, Franklin Lakes, NJ) antibodies. Quantification was performed as described [13] using Aperio Imagescope software. Approval for the acquisition of all human tissues was granted by the Institutional Review Board (IRB) at the Office of Research Administration at Indiana University.

### Immunoblotting and quantitative PCR

Immunoblotting was performed as described [65] using CD31 (BD Biosciences), phosphorylated and total SMAD, p-JAK2, PARP (Cell Signaling Technology, Danvers, MA), and p-JAK1, JAK1, JAK2 and ERK2 (Santa Cruz Biotechnology, Dallas, TX) antibodies. Briefly, lysates were prepared as described [65], and lysates from flash-frozen human PDAC tissues, and we prepared lysates from three different regions of each tumor. The three sets of lysates were immunoblotted separately. Quantification of band area in each immunoblot was performed using ImageJ software (http://imagej.nih.gov/ij/) from three independent experiments, and mean ± SEM. Quantitative PCR (qPCR) was performed for the indicated mRNAs as described [95] using RNA extracted from the same flash-frozen PDAC tissues. *RPS6* served as the endogenous control.

## SUPPLEMENTARY MATERIALS FIGURES AND TABLES



## Abbreviations

EC: Endothelial cell
GEMM: Genetically engineered mouse model
HUVEC: human vascular endothelial cells
JAK: Janus kinase
MVD: Microvessel density
PCC: Pancreatic cancer cell
PDAC: Pancreatic ductal adenocarcinoma
PNET: Pancreatic neuroendocrine tumor
TCGA: The Cancer Genome Atlas
TGF-β: Transforming growth factor-β
TβRI: Transforming growth factor-β type I receptor
TMA: Tissue microarray
TME: Tumor microenvironment
