# Supplementary material for: Angiogenic gene signature in human pancreatic cancer correlates with TGF-beta and inflammatory transcriptomes
Source: Oncotarget. 2015 Nov 18;7(1):323–41. doi: 10.18632/oncotarget.6345 (PMC4808001; doi:10.18632/oncotarget.6345)
Supplement: Supplementary file 1 [file oncotarget-07-0323-s001.pdf]

Angiogenic gene signature in human pancreatic cancer correlates with TGF-beta and inflammatory transcriptomes

Supplementary Materials

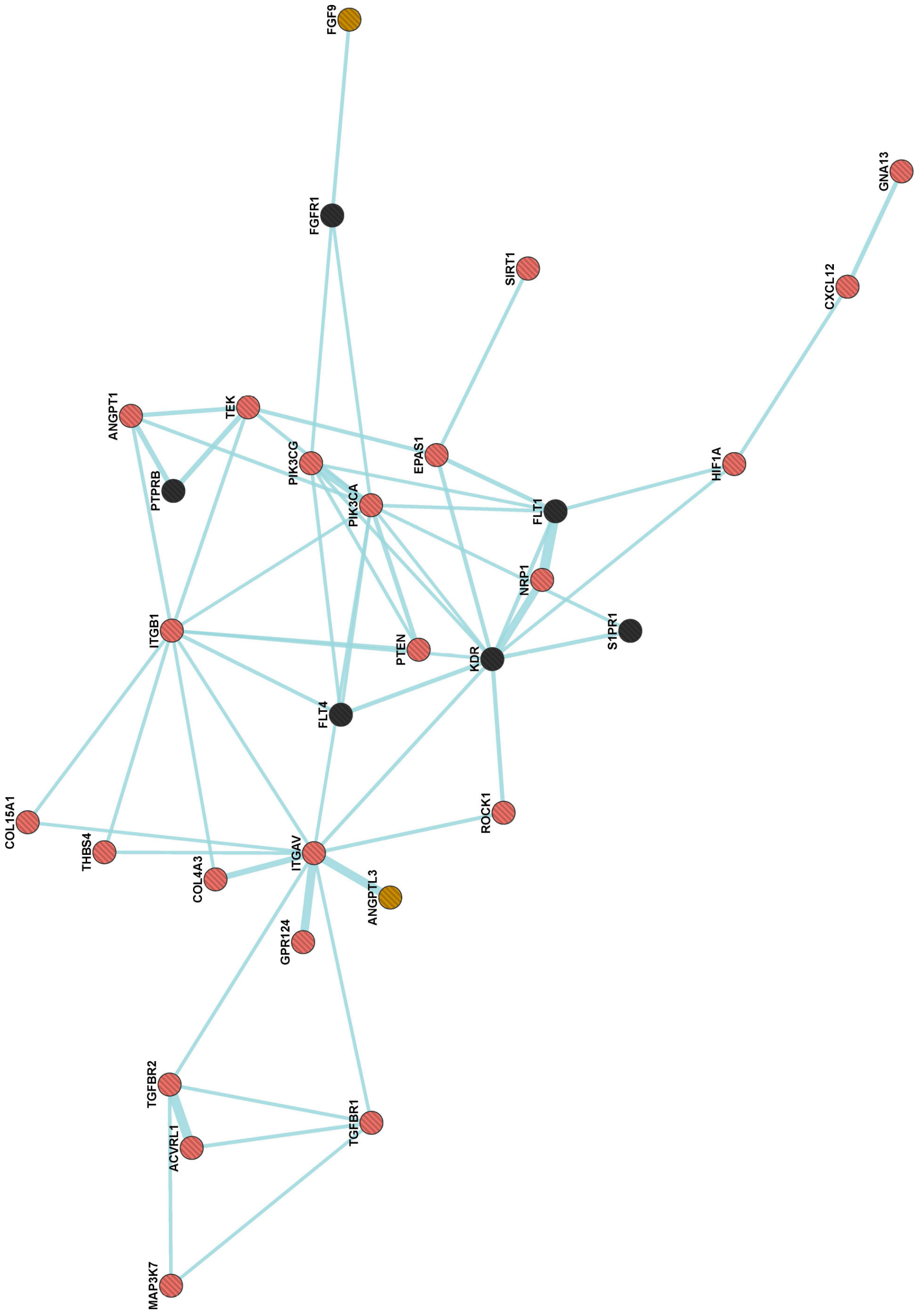

Supplementary Figure S1: Interconnectivity of angiogenesis genes in PDAC and PNET. GeneMANIA pathway analysis of the 48, 10, or 31 genes that are unique to the Strong Angiogenic PDAC group, unique to the PNET group, or common to both tumor types shows that 22 PDAC genes are interconnected (pink), whereas only two PNET genes are interconnected (mustard). 6 interconnected angiogenesis gene are common (black) and dominated by VEGF pathway members, while genes unique to PDAC (pink) also involve integrins, collagens and TGF-β related pathway members. Genes unique to PNET (mustard) do not have known pathway connections except for ANGPTL3 and FGF9.

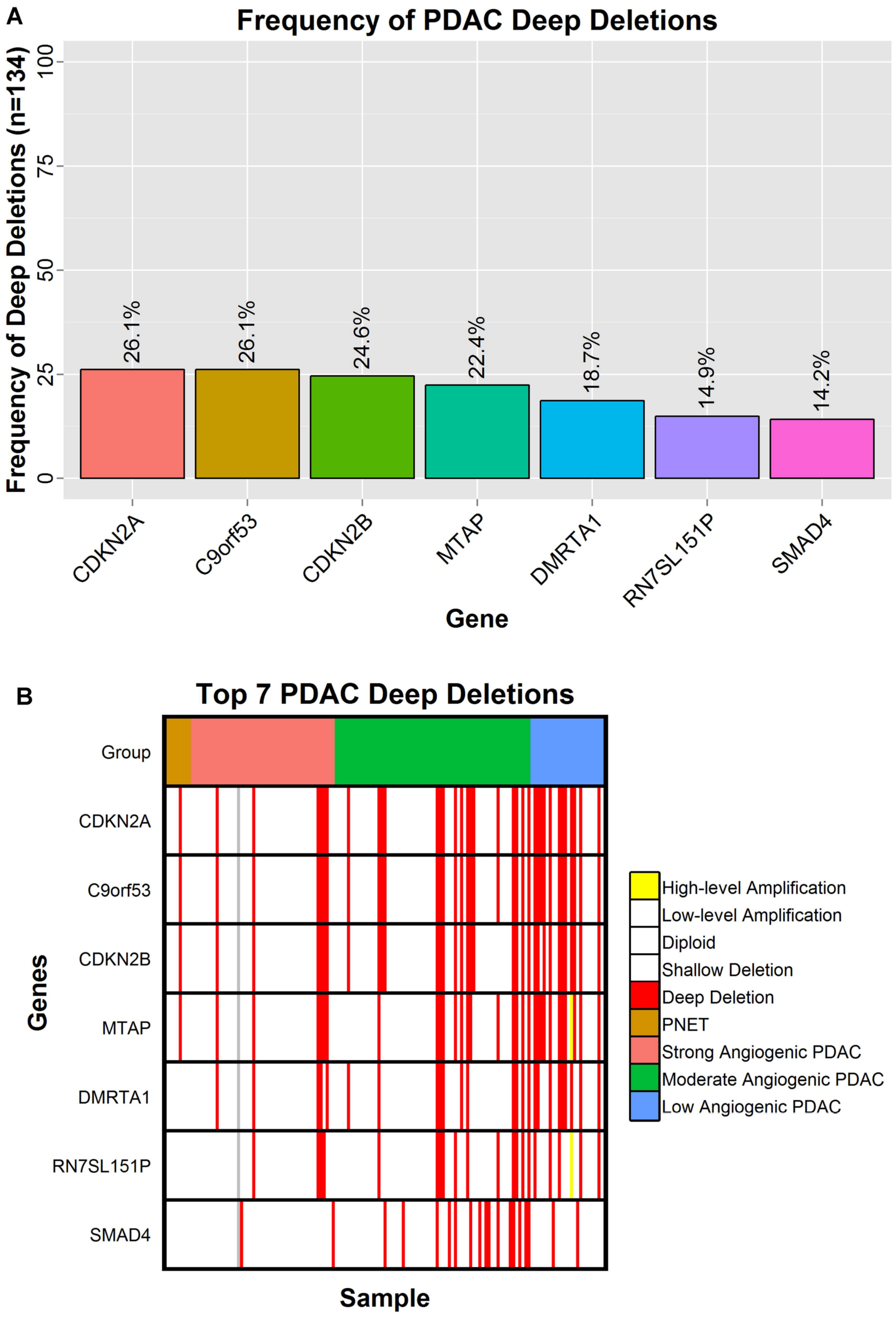

Supplementary Figure S2: Frequency and distribution of gene deletion in PDAC. (**A**) Frequency of the top 7 genes with deep deletions in PDAC samples from the TCGA. Frequency is shown as overall % of deletion in the 134 samples with copy number data. (**B**) Distribution of the top 7 deep deletions across the different PNET and PDAC angiogenic groups (8 PNET and 134 of 135 PDAC samples had copy number data. The missing PDAC sample is shown in gray). Several samples show deletion of many of these genes due to the top 6 genes appearing in the same cytoband.

Supplementary Table S1: Expression of 129 angiogenic genes in strong angiogenic PDAC and PNET vs. weak angiogenic PDAC

Entrez Gene ID	Gene Symbol	Strong Angiogenic PDAC vs. Weak Angiogenic PDAC			PNET vs. Weak Angiogenic PDAC		
		Fold Change	*P*-value	FDR	Fold Change	*P*-value	FDR
90	ACVR1	1.28	0.134419	0.283918	−1.55	0.053462	0.134242
94	ACVRL1	1.76	0.000104	0.000761	1.06	0.697914	0.84304
118	ADD1	1.20	0.276847	0.482664	1.42	0.093408	0.208904
55109	AGGF1	1.25	0.096651	0.220962	1.47	0.089227	0.201602
183	AGT	−1.21	0.824471	0.936687	37.33	0.085506	0.195078
284	ANGPT1	3.38	4.31E-08	8.07E-07	1.05	0.742976	0.876605
27329	ANGPTL3	1.15	0.92155	1	17.34	0.006417	0.024057
347	APOD	2.07	0.002255	0.01042	−4.75	0.000466	0.002631
350	APOH	1.57	0.725697	0.874353	42.65	0.018246	0.056763
81575	APOLD1	2.87	2.04E-09	5.67E-08	2.74	2.19E-05	0.000179
358	AQP1	1.34	0.017158	0.055445	−2.24	0.050883	0.129064
83478	ARHGAP24	1.31	0.035347	0.099629	−1.22	0.778738	0.904047
577	BAI3	4.25	0.016713	0.054225	47.69	2.18E-06	2.31E-05
168667	BMPER	4.36	0.001766	0.00849	12.06	6.04E-05	0.000439
718	C3	2.10	1.30E-05	0.000124	−9.90	5.24E-12	1.98E-10
719	C3AR1	3.23	2.08E-11	1.04E-09	−1.62	0.032684	0.091125
729	C6	2.32	0.005793	0.022837	−1.03	0.789036	0.911819
10203	CALCRL	4.15	3.29E-19	1.72E-16	1.91	0.001772	0.008227
729230	CCR2	5.70	3.24E-13	2.90E-11	−1.83	0.100532	0.220636
947	CD34	2.31	4.65E-09	1.15E-07	1.89	0.001753	0.008154
25932	CLIC4	2.75	5.92E-12	3.47E-10	−1.48	0.066506	0.159899
1215	CMA1	11.66	0.001092	0.005646	1.38	0.379612	0.586507
1306	COL15A1	3.20	2.23E-15	4.01E-13	−1.94	0.006385	0.023948
1285	COL4A3	3.72	0.001814	0.008666	1.11	0.824543	0.939269
1464	CSPG4	1.02	0.75723	0.893452	−1.31	0.447436	0.653026
1524	CX3CR1	2.85	6.95E-06	7.15E-05	2.29	0.014731	0.047777
6387	CXCL12	3.48	2.35E-11	1.16E-09	−1.29	0.983107	1
1545	CYP1B1	7.40	1.51E-29	1.50E-25	−1.37	0.095421	0.211965
57105	CYSLTR2	4.06	0.000438	0.002612	7.66	0.000206	0.001284
23405	DICER1	1.49	0.004659	0.019015	1.66	0.019014	0.058703
641700	ECSCR	1.69	0.004009	0.01686	1.42	0.179689	0.343343
54583	EGLN1	1.00	0.882916	0.976375	−1.16	0.580403	0.749433
2004	ELK3	2.62	1.16E-08	2.57E-07	−1.24	0.340933	0.54456

2028	ENPEP	2.73	2.38E-08	4.86E-07	1.66	0.04932	0.126023
2034	EPAS1	1.65	0.000206	0.001367	1.53	0.028162	0.080727
2047	EPHB1	3.18	0.043333	0.117391	2.63	0.231459	0.412084
51752	ERAP1	1.28	0.068527	0.16901	−1.11	0.624052	0.784646
356	FASLG	2.80	0.051675	0.135245	−1.52	0.642535	0.798728
2254	FGF9	1.94	0.06792	0.167857	3.84	0.006958	0.025646
2260	FGFR1	1.87	1.11E-06	1.43E-05	3.24	3.92E-10	1.02E-08
2263	FGFR2	−1.16	0.420471	0.640822	−17.60	3.68E-16	3.17E-14
2277	FIGF	3.59	0.018226	0.058344	3.28	0.06252	0.15215
2321	FLT1	2.11	1.30E-08	2.83E-07	3.36	1.04E-09	2.43E-08
2324	FLT4	2.46	1.25E-07	2.07E-06	3.77	2.77E-08	4.76E-07
51738	GHRL	1.41	0.288334	0.496086	−6.85	0.052634	0.132638
2702	GJA5	1.87	0.000107	0.000777	−1.03	0.829247	0.943158
10672	GNA13	1.73	0.000231	0.001498	−1.25	0.254681	0.441674
2822	GPLD1	3.50	0.006407	0.024772	21.29	1.37E-06	1.53E-05
25960	GPR124	2.89	1.72E-12	1.21E-10	1.69	0.01807	0.056321
26585	GREM1	3.82	0.00029	0.001829	−3.27	0.016599	0.05258
2969	GTF2I	1.37	0.039113	0.108126	2.22	9.34E-05	0.000647
9421	HAND1	42.99	0.256031	0.456074	26.50	0.196605	0.366146
9464	HAND2	2.28	0.000147	0.001022	1.43	0.330874	0.533066
9734	HDAC9	1.82	0.009783	0.034952	1.22	0.512487	0.707316
3087	HHEX	−1.02	0.837256	0.946165	−1.56	0.184215	0.349603
3091	HIF1A	2.01	2.14E-06	2.54E-05	−1.24	0.259919	0.447496
204851	HIPK1	1.63	0.000516	0.003004	1.19	0.388465	0.594742
28996	HIPK2	1.35	0.02309	0.070767	2.66	1.52E-06	1.67E-05
9394	HS6ST1	1.30	0.064347	0.160585	1.12	0.508225	0.705822
3553	IL1B	1.82	0.037926	0.105491	−1.44	0.366107	0.571362
3670	ISL1	1.69	0.178856	0.350616	19.37	1.82E-06	1.97E-05
3685	ITGAV	2.20	1.10E-07	1.86E-06	−2.45	4.19E-05	0.000321
3688	ITGB1	1.68	0.000343	0.002112	−1.41	0.101044	0.22149
83700	JAM3	1.75	0.000241	0.001559	1.61	0.02388	0.070574
3791	KDR	3.04	4.57E-14	5.61E-12	4.37	2.56E-13	1.29E-11
11061	LECT1	1.38	0.560306	0.772961	1.65	0.554378	0.730645
6885	MAP3K7	1.57	0.004483	0.018433	1.10	0.663338	0.814751
4162	MCAM	1.96	2.13E-06	2.54E-05	1.74	0.011287	0.038288
5469	MED1	1.33	0.073878	0.178863	1.19	0.558488	0.733027
4223	MEOX2	3.63	2.35E-08	4.81E-07	−1.08	0.861664	0.966734
79812	MMRN2	2.25	2.82E-08	5.62E-07	1.79	0.003774	0.015521
92140	MTDH	1.34	0.036302	0.101787	−1.21	0.358553	0.563493

80155	NAA15	1.18	0.212152	0.398612	−1.16	0.530401	0.713916
4763	NF1	1.39	0.022444	0.069223	1.47	0.103588	0.225862
4775	NFATC3	1.22	0.140951	0.2946	−1.64	0.064087	0.155272
4881	NPR1	2.48	2.86E-08	5.69E-07	2.63	2.71E-05	0.000217
4897	NRCAM	2.39	1.15E-05	0.000112	13.16	2.44E-15	1.74E-13
8829	NRP1	2.38	8.17E-10	2.59E-08	1.32	0.111065	0.238476
9378	NRXN1	4.30	5.12E-06	5.47E-05	20.78	3.32E-12	1.31E-10
9369	NRXN3	3.83	0.000877	0.004694	12.05	7.04E-06	6.57E-05
116150	NUS1	1.29	0.043239	0.117168	1.03	0.757191	0.887489
55742	PARVA	1.36	0.025279	0.076065	−2.06	0.004041	0.016436
5140	PDE3B	2.34	0.004105	0.017186	5.72	2.92E-05	0.000232
5290	PIK3CA	2.24	1.89E-06	2.27E-05	−1.28	0.31137	0.510688
5294	PIK3CG	5.16	1.46E-12	1.06E-10	−2.09	0.010269	0.035493
5316	PKNOX1	1.13	0.400487	0.619976	1.14	0.441351	0.647094
57125	PLXDC1	1.95	3.71E-05	0.000311	1.53	0.138244	0.281608
23129	PLXND1	1.63	0.001399	0.006985	1.27	0.36716	0.572789
23509	POFUT1	1.24	0.100837	0.228037	1.53	0.041843	0.111043
5578	PRKCA	1.03	1	1	−1.81	0.004233	0.017093
5613	PRKX	1.35	0.076365	0.183542	−3.49	1.54E-05	0.000132
84432	PROK1	4.43	0.110425	0.244269	−1.46	1	1
60675	PROK2	2.95	0.433556	0.654636	−1.97	0.831581	0.94463
5728	PTEN	1.57	0.000789	0.004287	−1.04	0.943968	1
5787	PTPRB	2.19	1.37E-07	2.22E-06	2.66	1.21E-05	0.000106
5797	PTPRM	1.76	8.35E-05	0.00063	2.64	8.02E-07	9.52E-06
10266	RAMP2	1.50	0.003989	0.016798	1.83	0.004153	0.016826
6091	ROBO1	3.82	1.68E-16	4.23E-14	−1.17	0.463909	0.668963
54538	ROBO4	1.97	6.59E-06	6.84E-05	2.79	2.22E-06	2.35E-05
6093	ROCK1	1.67	0.001488	0.007347	−1.09	0.542624	0.722145
9475	ROCK2	1.74	0.000324	0.002018	−1.49	0.081406	0.187464
6095	RORA	2.53	4.57E-09	1.13E-07	1.88	0.004834	0.019105
1901	S1PR1	3.52	2.73E-16	6.45E-14	1.65	0.013022	0.043103
23328	SASH1	2.69	1.71E-10	6.53E-09	2.27	1.61E-05	0.000137
7857	SCG2	3.51	0.057532	0.147316	65.37	7.42E-06	6.87E-05
9723	SEMA3E	3.64	0.000257	0.001651	4.75	0.001244	0.006095
29072	SETD2	1.32	0.051775	0.135422	1.22	0.301772	0.499009
6422	SFRP1	9.57	4.36E-07	6.22E-06	29.50	1.53E-09	3.46E-08
23411	SIRT1	1.67	0.001562	0.00765	1.24	0.358061	0.562989
9990	SLC12A6	1.42	0.038293	0.10623	−1.08	0.662316	0.81414
9353	SLIT2	8.61	1.44E-22	1.90E-19	2.52	0.002719	0.011783

6722	SRF	1.15	0.357784	0.575258	−1.12	0.67388	0.823416
6733	SRPK2	1.49	0.006965	0.026514	2.14	0.00067	0.00359
23166	STAB1	2.29	2.32E-08	4.76E-07	1.19	0.527104	0.712285
6886	TAL1	2.52	0.000161	0.001104	2.60	0.004552	0.018169
7010	TEK	3.74	1.13E-14	1.57E-12	1.65	0.026744	0.077372
7046	TGFBR1	1.53	0.00904	0.032787	−1.02	0.751031	0.883048
7048	TGFBR2	1.86	4.36E-05	0.000356	−1.56	0.018017	0.056204
7060	THBS4	5.55	7.95E-11	3.27E-09	1.98	0.068252	0.163316
221981	THSD7A	4.72	7.58E-11	3.14E-09	−1.08	0.708204	0.850732
7075	TIE1	2.21	1.46E-07	2.35E-06	1.58	0.026074	0.075776
55273	TMEM100	3.80	4.42E-05	0.00036	5.52	0.000318	0.001886
164656	TMPRSS6	−1.49	0.641486	0.833109	23.17	0.02359	0.069884
23554	TSPAN12	1.00	0.885861	0.978542	3.37	0.000204	0.001275
7342	UBP1	1.19	0.231426	0.42474	−1.16	0.564326	0.736234
22846	VASH1	2.06	1.72E-06	2.10E-05	3.61	2.33E-09	5.06E-08
10451	VAV3	1.56	0.068866	0.169688	2.21	0.035368	0.09703
7716	VEZF1	1.33	0.033594	0.095708	1.73	0.007181	0.026347
10163	WASF2	1.58	0.001278	0.006463	−1.17	0.490966	0.692833

PDAC: Pancreatic Ductal Adenocarcinoma; PNET: Pancreatic Neuroendocrine Tumor; FDR: False Discovery Rate; 31 genes up-regulated in both PDAC and PNET vs. Weak Angiogenic PDAC; 79 differentially expressed genes in Strong Angiogenic PDAC vs. Weak Angiogenic PDAC (79 up-regulated, 0 down-regulated); 50 differentially expressed genes in PNET vs. Weak Angiogenic PDAC (41 up-regulated, 9 down-regulated); Differential expression cut off: |Fold Change| > = 1.5, FDR < 0.05.

Supplementary Table S2: Mutation frequency in PNET and PDAC angiogenic groups

Gene Symbol	Total # Mutated	Total # Sequenced	Total % Mutated	PNET # Mutated	PNET # Sequenced	PNET % Mutated	PDAC # Mutated	PDAC # Sequenced	PDAC % Mutated	Strong Angiogenic PDAC # Mutated	Strong Angiogenic PDAC # Sequenced	Strong Angiogenic PDAC % Mutated	Moderate Angiogenic PDAC # Mutated	Moderate Angiogenic PDAC # Sequenced	Moderate Angiogenic PDAC % Mutated	Weak Angiogenic PDAC # Mutated	Weak Agniogenic PDAC # Sequenced	Weak Angiogenic PDAC % Mutated
KRAS	84	101	83.17	0	3	0.00	84	98	85.71	33	41	80.49	36	38	94.74	15	19	78.95
TP53	54	101	53.47	0	3	0.00	54	98	55.10	19	41	46.34	26	38	68.42	9	19	47.37
TTN	20	101	19.80	0	3	0.00	20	98	20.41	8	41	19.51	6	38	15.79	6	19	31.58
SMAD4	19	101	18.81	0	3	0.00	19	98	19.39	4	41	9.76	9	38	23.68	6	19	31.58
CDKN2A	19	101	18.81	0	3	0.00	19	98	19.39	2	41	4.88	14	38	36.84	3	19	15.79
MUC16	10	101	9.90	0	3	0.00	10	98	10.20	4	41	9.76	3	38	7.89	3	19	15.79
SPTA1	9	101	8.91	0	3	0.00	9	98	9.18	3	41	7.32	4	38	10.53	2	19	10.53
RGPD3	9	101	8.91	0	3	0.00	9	98	9.18	4	41	9.76	4	38	10.53	1	19	5.26
LRP1B	9	101	8.91	0	3	0.00	9	98	9.18	3	41	7.32	3	38	7.89	3	19	15.79
FAT3	9	101	8.91	0	3	0.00	9	98	9.18	2	41	4.88	4	38	10.53	3	19	15.79
ANAPC1	9	101	8.91	0	3	0.00	9	98	9.18	2	41	4.88	4	38	10.53	3	19	15.79
KMT2D	8	101	7.92	2	3	66.67	6	98	6.12	2	41	4.88	2	38	5.26	2	19	10.53
RYR1	7	101	6.93	0	3	0.00	7	98	7.14	2	41	4.88	3	38	7.89	2	19	10.53
RNF43	7	101	6.93	0	3	0.00	7	98	7.14	1	41	2.44	3	38	7.89	3	19	15.79
PLEC	7	101	6.93	0	3	0.00	7	98	7.14	2	41	4.88	3	38	7.89	2	19	10.53
OBSCN	7	101	6.93	0	3	0.00	7	98	7.14	3	41	7.32	2	38	5.26	2	19	10.53
KBTBD6	7	101	6.93	1	3	33.33	6	98	6.12	0	41	0.00	1	38	2.63	5	19	26.32
HMCN1	7	101	6.93	0	3	0.00	7	98	7.14	3	41	7.32	1	38	2.63	3	19	15.79
CSMD2	7	101	6.93	0	3	0.00	7	98	7.14	3	41	7.32	2	38	5.26	2	19	10.53
USH2A	6	101	5.94	0	3	0.00	6	98	6.12	3	41	7.32	2	38	5.26	1	19	5.26
TENM3	6	101	5.94	0	3	0.00	6	98	6.12	1	41	2.44	4	38	10.53	1	19	5.26
SPATA31D1	6	101	5.94	0	3	0.00	6	98	6.12	3	41	7.32	2	38	5.26	1	19	5.26

RYR3	6	101	5.94	0	3	0.00	6	98	6.12	4	41	9.76	2	38	5.26	0	19	0.00
PCDH15	6	101	5.94	0	3	0.00	6	98	6.12	1	41	2.44	2	38	5.26	3	19	15.79
MYH4	6	101	5.94	0	3	0.00	6	98	6.12	2	41	4.88	2	38	5.26	2	19	10.53
KCNA6	6	101	5.94	0	3	0.00	6	98	6.12	3	41	7.32	2	38	5.26	1	19	5.26
HERC2	6	101	5.94	0	3	0.00	6	98	6.12	5	41	12.20	1	38	2.63	0	19	0.00
GLI3	6	101	5.94	0	3	0.00	6	98	6.12	2	41	4.88	1	38	2.63	3	19	15.79
FLNA	6	101	5.94	1	3	33.33	5	98	5.10	3	41	7.32	2	38	5.26	0	19	0.00
FAT4	6	101	5.94	0	3	0.00	6	98	6.12	4	41	9.76	2	38	5.26	0	19	0.00
CELSR3	6	101	5.94	0	3	0.00	6	98	6.12	2	41	4.88	2	38	5.26	2	19	10.53
BTBD11	6	101	5.94	0	3	0.00	6	98	6.12	1	41	2.44	2	38	5.26	3	19	15.79
ARID1A	6	101	5.94	0	3	0.00	6	98	6.12	3	41	7.32	1	38	2.63	2	19	10.53
ADAMTS12	6	101	5.94	0	3	0.00	6	98	6.12	3	41	7.32	2	38	5.26	1	19	5.26

PNET: Pancreatic Neuroendocrine Tumor, PDAC: Pancreatic Ductal Adenocarcinoma, #: Number, %: Percent

Supplementary Table S3: Expression of 186 TGF-β responsive genes in strong angiogenic PDAC and PNET vs. weak angiogenic PDAC

Entrez Gene ID	Gene Symbol	Strong Angiogenic PDAC vs. Weak Angiogenic PDAC			PNET vs. Weak Angiogenic PDAC		
		Fold Change	*P*-value	FDR	Fold Change	*P*-value	FDR
25841	ABTB2	−1.50	0.001686	0.008159	−8.76	6.63E-13	3.06E-11
23205	ACSBG1	1.23	1	1	24.25	0.175672	0.337868
53616	ADAM22	7.97	3.42E-13	3.01E-11	3.78	0.000637	0.003431
9590	AKAP12	3.79	1.37E-10	5.31E-09	1.62	0.198132	0.368052
286	ANK1	−2.98	0.000112	0.000807	1.22	0.660044	0.812612
23141	ANKLE2	−1.24	0.0763	0.183431	1.13	0.631461	0.790037
94134	ARHGAP12	−1.20	0.180306	0.35276	−1.92	0.006344	0.02383
467	ATF3	−1.16	0.280025	0.485873	−2.58	0.02225	0.066527
54880	BCOR	1.00	0.925665	1	1.70	0.007745	0.028063
8553	BHLHE40	−1.44	0.001574	0.007694	−3.61	5.50E-08	8.81E-07
635	BHMT	−1.27	0.674828	0.850796	1.58	0.677618	0.826618
659	BMPR2	1.93	1.26E-05	0.000121	−1.06	0.710588	0.852558
676	BRDT	3.41	1	1	−2.19	1	1
28984	C13orf15	1.56	0.02266	0.069772	1.90	0.019646	0.060253
126353	C19orf21	−3.93	2.34E-17	7.37E-15	−6.85	9.82E-10	2.30E-08
27042	C1orf107	−1.05	0.693799	0.857321	−2.17	0.001376	0.006637
81626	C1orf14	1.78	1	1	-Inf	1	1
221749	C6orf145	1.13	0.483499	0.704075	−2.07	0.001577	0.007445
203197	C9orf91	1.53	0.006379	0.024712	1.78	0.014934	0.04824
65981	CAPRIN2	−2.34	0.000191	0.001277	−1.61	0.25224	0.438729
838	CASP5	1.26	0.770198	0.901502	−5.72	0.374824	0.58161
79780	CCDC82	1.14	0.518067	0.737443	−1.22	0.480953	0.684663
54520	CCDC93	1.15	0.355736	0.573358	1.34	0.1724	0.332867
9332	CD163	5.32	3.03E-11	1.42E-09	−1.99	0.037955	0.102589
913	CD1E	3.58	9.66E-05	0.000714	−2.72	0.024562	0.072198
10849	CD3EAP	−1.01	0.766337	0.899805	−1.30	0.255079	0.441977
5128	CDK17	1.68	0.001242	0.006311	1.42	0.09233	0.207009
55602	CDKN2AIP	−1.26	0.089821	0.208515	1.10	0.696386	0.841997
1030	CDKN2B	1.82	0.013022	0.044207	−3.29	0.000765	0.004013
50515	CHST11	1.82	0.001907	0.009035	−1.76	0.01087	0.037173
9469	CHST3	1.90	0.000113	0.000811	−1.02	0.710898	0.852776
1160	CKMT2	1.08	0.683904	0.853518	−3.48	0.0181	0.056383
23529	CLCF1	−1.42	0.009147	0.033102	−18.01	1.68E-18	1.96E-16

1307	COL16A1	1.39	0.044775	0.120671	−2.57	0.000189	0.001196
1410	CRYAB	1.57	0.27534	0.480768	3.35	0.038759	0.104317
1436	CSF1R	3.09	2.65E-13	2.47E-11	−1.23	0.247224	0.432026
1454	CSNK1E	−1.38	0.011202	0.039043	−1.04	0.835104	0.947254
1474	CST6	−1.35	0.247021	0.445257	−116.94	8.77E-08	1.33E-06
1490	CTGF	3.33	1.11E-07	1.86E-06	1.03	0.862929	0.967639
1591	CYP24A1	1.09	1	1	−100.23	0.008872	0.031411
56603	CYP26B1	2.77	8.09E-05	0.000614	2.33	0.016291	0.05174
1573	CYP2J2	−2.24	2.89E-06	3.28E-05	−6.82	1.27E-08	2.36E-07
9267	CYTH1	−1.05	0.354144	0.571534	−2.02	0.003228	0.013609
51339	DACT1	2.63	4.39E-09	1.10E-07	−2.28	0.00157	0.007418
1644	DDC	−1.90	0.396732	0.61618	15.03	0.002554	0.011166
25786	DGCR11	−1.14	0.652232	0.839763	−1.74	0.313331	0.512871
10395	DLC1	3.17	6.90E-14	7.65E-12	2.19	0.000228	0.001405
23333	DPY19L1	1.03	1	1	−1.94	0.003275	0.013794
8445	DYRK2	1.28	0.092332	0.212974	−1.09	0.658264	0.811179
1906	EDN1	1.12	0.467069	0.68813	−4.87	3.04E-07	4.03E-06
22936	ELL2	1.79	4.12E-06	4.50E-05	7.23	6.82E-24	2.01E-21
2043	EPHA4	1.03	0.812846	0.929315	1.48	0.217778	0.394668
2114	ETS2	1.10	0.687442	0.85424	−2.05	0.000467	0.002634
2150	F2RL1	−1.65	0.00474	0.01931	−8.60	1.88E-10	5.23E-09
79633	FAT4	5.32	1.58E-20	1.25E-17	2.02	0.007414	0.027068
10979	FERMT2	3.11	1.15E-13	1.17E-11	1.29	0.235036	0.416798
2244	FGB	−1.41	0.724069	0.873385	116.69	0.098781	0.217665
2246	FGF1	1.91	0.194811	0.374355	12.11	4.87E-05	0.000365
54874	FNBP1L	1.10	0.463622	0.684982	1.20	0.381186	0.588152
2297	FOXD1	−2.96	7.67E-05	0.000585	−21.06	6.94E-06	6.49E-05
2295	FOXF2	1.21	0.462769	0.684307	−2.33	0.005274	0.020498
51343	FZR1	−1.78	8.28E-06	8.38E-05	1.28	0.310171	0.509018
4616	GADD45B	1.31	0.244027	0.441464	1.16	0.383587	0.590564
8843	GPR109B	2.24	0.063272	0.15848	−5.38	0.008142	0.029254
3142	HLX	1.21	0.418067	0.638368	−1.25	0.273136	0.463779
3269	HRH1	−1.23	0.078402	0.18734	−4.27	4.77E-08	7.75E-07
9956	HS3ST2	1.95	0.240568	0.43662	−5.09	0.059959	0.147315
3352	HTR1D	−1.06	0.843386	0.949958	−5.50	0.000507	0.002821
3373	HYAL1	−2.82	3.10E-06	3.49E-05	−9.79	1.29E-06	1.45E-05
3399	ID3	1.03	0.898439	0.986619	−1.06	0.584586	0.752716
3589	IL11	1.87	0.48517	0.705724	−4.52	0.219862	0.397423
29949	IL19	−2.29	0.346467	0.563436	-Inf	0.235528	0.417483

9173	IL1RL1	3.88	0.004441	0.01831	1.97	0.26266	0.45084
3667	IRS1	1.30	0.164925	0.330817	−1.55	0.018936	0.058514
3678	ITGA5	1.88	0.00143	0.007113	−1.76	0.025214	0.073721
3694	ITGB6	−1.20	0.196205	0.376223	−156.01	5.74E-19	7.26E-17
58494	JAM2	2.83	2.32E-08	4.76E-07	1.24	0.307727	0.505975
221037	JMJD1C	1.57	0.003792	0.016096	−1.08	0.663027	0.814641
23210	JMJD6	−1.20	0.141488	0.29553	1.21	0.471906	0.676124
3725	JUN	−1.11	0.230011	0.423357	−1.66	0.010102	0.034982
3726	JUNB	−1.75	1.27E-06	1.61E-05	−1.67	0.010997	0.037534
23189	KANK1	1.52	0.013635	0.045944	3.10	3.24E-06	3.28E-05
3783	KCNN4	−3.25	4.45E-15	7.31E-13	−163.87	3.09E-33	3.05E-30
23135	KDM6B	1.21	0.263495	0.46611	1.85	0.005399	0.020896
80759	KHDC1	−1.36	0.302388	0.512847	2.34	0.068656	0.164048
7071	KLF10	1.51	0.008035	0.029789	1.09	0.599354	0.765094
9314	KLF4	−1.66	0.000337	0.002085	−3.89	2.54E-06	2.64E-05
8609	KLF7	2.08	0.000897	0.004785	−1.32	0.200868	0.371979
55323	LARP6	1.09	0.671374	0.849771	1.29	0.355642	0.560123
23592	LEMD3	1.34	0.040008	0.110186	1.27	0.257506	0.444505
3976	LIF	−1.36	0.041359	0.113	−3.12	7.30E-05	0.000522
9388	LIPG	1.82	0.001285	0.006494	−2.10	0.003518	0.014642
29995	LMCD1	1.73	0.001973	0.009297	−1.03	0.801165	0.921573
29967	LRP12	3.39	3.32E-12	2.14E-10	3.33	4.17E-07	5.30E-06
4053	LTBP2	3.07	6.28E-14	7.21E-12	−1.12	0.383343	0.590308
4054	LTBP3	1.12	0.530327	0.747979	1.51	0.038431	0.103591
116372	LYPD1	1.03	0.866997	0.965274	−3.04	0.014884	0.048133
9935	MAFB	2.69	0.008175	0.030182	17.59	1.58E-08	2.86E-07
4216	MAP3K4	1.42	0.014183	0.047443	1.87	0.003287	0.01383
4142	MAS1	1.42	1	1	129.46	0.241457	0.425281
4146	MATN1	1.22	0.978986	1	2.00	0.557309	0.732503
54797	MED18	−1.22	0.335781	0.552403	−1.34	0.375345	0.582188
57591	MKL1	1.06	0.967787	1	−1.60	0.046285	0.120024
22877	MLXIP	1.65	0.000496	0.002906	1.89	0.002791	0.01205
23531	MMD	1.87	9.99E-05	0.000734	1.05	0.880584	0.979094
10205	MPZL2	−1.15	0.368703	0.586358	−12.34	4.50E-12	1.73E-10
9617	MTRF1	−1.38	0.067629	0.167219	−1.27	0.400481	0.606918
4606	MYBPC2	3.79	0.152071	0.311643	−7.54	0.246888	0.43161
9612	NCOR2	−1.17	0.132268	0.280335	1.73	0.011355	0.03845
3340	NDST1	1.87	2.48E-06	2.88E-05	3.53	4.37E-11	1.39E-09
4776	NFATC4	1.04	0.859542	0.959934	1.66	0.168547	0.326941

4803	NGF	1.62	0.27086	0.475285	1.04	0.722244	0.861361
4908	NTF3	1.15	0.882171	0.97594	−1.29	0.852683	0.960022
58495	OVOL2	−3.37	1.75E-12	1.22E-10	−2.35	0.01552	0.049757
400961	PAIP2B	−1.41	0.761897	0.897157	3.31	0.0123	0.04122
5154	PDGFA	−1.67	0.0003	0.001883	−1.31	0.568087	0.739334
5155	PDGFB	1.05	1	1	−1.25	0.25153	0.437865
8572	PDLIM4	−1.04	0.592905	0.797824	1.81	0.134812	0.276712
5209	PFKFB3	1.14	0.837375	0.946192	−1.26	0.279043	0.471457
9767	PHF16	1.15	0.320814	0.535408	−1.10	0.903682	0.995004
23187	PHLDB1	1.51	0.004391	0.018137	−1.88	0.003485	0.014525
5293	PIK3CD	2.81	1.21E-08	2.66E-07	−1.66	0.040039	0.107091
5569	PKIA	1.80	0.007294	0.027533	2.28	0.006303	0.023725
59338	PLEKHA1	−1.13	0.373285	0.591042	−1.27	0.276309	0.467919
56937	PMEPA1	−1.15	0.274573	0.479725	−9.24	6.06E-13	2.83E-11
5376	PMP22	2.19	0.000293	0.001847	−1.47	0.182326	0.347239
5467	PPARD	−1.02	0.628708	0.824119	−2.12	0.00119	0.00587
10848	PPP1R13L	−2.53	1.42E-10	5.51E-09	−5.89	2.83E-09	6.02E-08
5521	PPP2R2B	2.29	0.001532	0.007525	13.74	9.09E-13	4.06E-11
5553	PRG2	−1.68	0.299683	0.509526	−2.08	0.496087	0.69676
79037	PVRIG	2.06	0.095408	0.218622	1.47	0.456456	0.661672
9693	RAPGEF2	1.43	0.015956	0.052179	1.43	0.124769	0.260478
23186	RCOR1	−1.24	0.112413	0.247689	−1.06	0.742573	0.876287
8786	RGS11	−1.46	0.199496	0.380632	6.38	1.66E-05	0.00014
6002	RGS12	−1.94	2.18E-07	3.35E-06	1.20	0.625767	0.786101
388	RHOB	−1.32	0.005838	0.022959	−1.56	0.019829	0.060644
6038	RNASE4	−1.58	0.033794	0.096078	1.73	0.072076	0.170564
376412	RNF126P1	−1.94	0.404669	0.624551	−2.36	0.86816	0.970968
127544	RNF19B	1.01	0.818081	0.932219	−1.18	0.437159	0.643217
6236	RRAD	1.16	0.998934	1	−2.56	0.650931	0.805719
9853	RUSC2	1.11	0.73376	0.879502	1.35	0.41221	0.618671
6274	S100A3	−1.57	0.045352	0.121858	−5.19	0.000364	0.002116
29970	SCHIP1	1.64	0.00758	0.028386	2.28	0.001727	0.008049
23541	SEC14L2	−1.30	0.08198	0.193917	−3.12	0.00024	0.001468
6446	SGK1	2.19	1.45E-08	3.12E-07	−1.82	0.022461	0.067054
8631	SKAP1	−2.36	0.155027	0.316038	3.29	0.103478	0.225697
6498	SKIL	1.99	4.10E-05	0.000338	−3.04	3.11E-06	3.17E-05
10786	SLC17A3	−1.22	0.770785	0.901891	3.82	0.482116	0.685895
1836	SLC26A2	2.44	3.83E-09	9.75E-08	2.37	5.86E-05	0.000428
9057	SLC7A6	1.47	0.01728	0.055779	−1.17	0.51088	0.707316

6578	SLCO2A1	1.53	0.025653	0.076978	1.14	0.625347	0.785874
4091	SMAD6	−2.33	1.92E-07	3.01E-06	−2.97	0.000274	0.001651
54498	SMOX	−2.24	2.85E-08	5.68E-07	−3.86	1.03E-07	1.53E-06
6525	SMTN	1.26	0.710213	0.866357	−2.09	0.315032	0.514662
57154	SMURF1	−1.50	0.000728	0.004008	−1.87	0.014587	0.047364
64750	SMURF2	1.56	0.005722	0.02259	−1.12	0.641038	0.797712
6615	SNAI1	2.11	5.14E-05	0.000412	−1.92	0.016928	0.05343
9306	SOCS6	1.34	0.057184	0.146596	1.66	0.03024	0.085499
8877	SPHK1	−1.35	0.053592	0.139147	−5.92	9.06E-08	1.36E-06
80176	SPSB1	1.73	0.000173	0.001177	−1.81	0.00912	0.032157
23648	SSBP3	−1.32	0.062633	0.157215	1.20	0.185921	0.351489
6781	STC1	2.20	9.74E-07	1.26E-05	2.02	0.003392	0.0142
6862	T	−3.83	0.419899	0.64036	−2.70	1	1
6876	TAGLN	1.83	0.012635	0.043109	−2.38	0.021745	0.065283
23102	TBC1D2B	1.60	0.001821	0.008694	−1.19	0.411877	0.618354
9519	TBPL1	1.08	0.560233	0.772924	1.46	0.109594	0.235857
6926	TBX3	1.90	0.000278	0.001766	1.91	0.040844	0.108845
7942	TFEB	−1.17	0.203398	0.386332	1.35	0.244926	0.429114
7066	THPO	−1.09	1	1	−5.55	0.12764	0.265208
7090	TLE3	−1.18	0.138481	0.290477	−1.25	0.504674	0.703056
79905	TMC7	−2.69	6.89E-11	2.90E-09	−22.60	5.03E-20	7.63E-18
25816	TNFAIP8	1.55	0.012616	0.043051	−6.96	1.44E-11	4.98E-10
84951	TNS4	−1.83	0.010356	0.03666	−2250.70	7.26E-16	5.78E-14
10221	TRIB1	−1.10	0.277087	0.482704	−2.46	0.000124	0.000823
10382	TUBB4	−1.28	0.283641	0.49036	−1.37	0.392716	0.599197
56995	TULP4	1.39	0.026084	0.077982	1.70	0.019603	0.06014
5412	UBL3	1.36	0.020416	0.064146	2.59	7.10E-06	6.61E-05
7481	WNT11	−2.26	0.047416	0.126231	−6.39	0.077876	0.18138
51384	WNT16	−1.76	0.225762	0.417354	−1.34	0.782576	0.907168
7473	WNT3	−1.02	0.931365	1	1.19	0.821232	0.937107
54361	WNT4	1.24	0.589148	0.794771	30.49	6.42E-07	7.81E-06
7476	WNT7A	−2.30	0.000805	0.004364	−94.33	1.26E-11	4.41E-10
81555	YIPF5	1.35	0.024842	0.074982	1.22	0.352082	0.556469
23174	ZCCHC14	1.10	0.562568	0.774584	1.05	0.763676	0.892619
677	ZFP36L1	1.33	0.083793	0.197238	−4.82	5.16E-11	1.62E-09
55279	ZNF654	1.35	0.103117	0.231715	−1.03	0.90664	0.997089

PDAC: Pancreatic Ductal Adenocarcinoma; PNET: Pancreatic Neuroendocrine Tumor; FDR: False Discovery Rate; 15 genes up-regulated in both PDAC and PNET vs. Weak Angiogenic PDAC; 50 genes up-regulated in Strong Angiogenic PDAC vs. Weak Angiogenic PDAC; 25 genes up-regulated in PNET vs. Weak Angiogenic PDAC; Differential expression cut off: Fold Change > = 1.5, FDR < 0.05.

Supplementary Table S4: Expression of 81 negative regulators of inflammation in strong angiogenic PDAC vs. weak angiogenic PDAC

Entrez Gene ID	Gene Symbol	Strong Angiogenic PDAC vs. Weak Angiogenic PDAC		
		Fold Change	*P*-value	FDR
29	ABR	−1.02	0.827246	0.938538
54	ACP5	1.29	0.613756	0.813698
100	ADA	1.16	0.487718	0.708096
116	ADCYAP1	3.88	0.001388	0.006933
9370	ADIPOQ	10.99	0.003206	0.013933
134	ADORA1	−1.44	0.347902	0.56478
325	APCS	1.03	0.807491	0.925483
335	APOA1	1.02	1	1
348	APOE	1.18	0.912798	0.99624
55870	ASH1L	1.38	0.022006	0.068192
613	BCR	−1.51	0.001573	0.007693
114899	C1QTNF3	2.14	0.020014	0.063193
80381	CD276	1.07	0.822491	0.93528
66005	CHID1	−1.45	0.077573	0.185794
1269	CNR2	26.26	0.000343	0.002114
11221	DUSP10	−1.32	0.116857	0.255058
1991	ELANE	−1.02	0.767792	0.900335
90268	FAM105B	1.16	0.368931	0.586627
388581	FAM132A	−3.37	3.10E-05	0.000265
55527	FEM1A	−1.03	1	1
2294	FOXF1	2.12	0.000595	0.003377
50943	FOXP3	4.20	1.38E-06	1.73E-05
2625	GATA3	1.50	0.328561	0.544631
2629	GBA	−1.17	0.335523	0.55207
2693	GHSR	4.83	0.371577	0.589423
2852	GPER	−1.54	0.580602	0.788616
338557	GPR120	−4.11	0.028049	0.082564
2876	GPX1	−1.61	0.000157	0.001084
2877	GPX2	−3.32	9.25E-08	1.60E-06
2950	GSTP1	−2.32	5.91E-11	2.53E-09
3123	HLA-DRB1	2.08	0.000373	0.00227
8870	IER3	−2.53	7.66E-08	1.34E-06
3586	IL10	3.03	0.035074	0.099084

3593	IL12B	3.16	0.334213	0.550746
3558	IL2	2.88	0.772575	0.902868
53833	IL20RB	−1.47	0.330891	0.547291
116379	IL22RA2	1.01	0.799682	0.920372
3559	IL2RA	3.84	3.93E-08	7.47E-07
3565	IL4	1.45	0.97783	1
3630	INS	1.18	0.794523	0.917077
9314	KLF4	−1.66	0.000337	0.002085
3848	KRT1	4.36	0.072277	0.176083
5598	MAPK7	−1.03	0.579672	0.787785
4210	MEFV	3.99	0.003634	0.015501
284207	METRNL	−1.50	0.000295	0.001854
4598	MVK	−1.87	9.38E-05	0.000697
80762	NDFIP1	1.00	0.721453	0.872355
4790	NFKB1	1.28	0.093579	0.215251
91662	NLRP12	2.60	0.072132	0.175901
114548	NLRP3	3.99	6.42E-09	1.53E-07
79671	NLRX1	−1.24	0.11242	0.247689
64127	NOD2	1.23	0.601968	0.804244
10062	NR1H3	−1.55	0.000785	0.004272
4907	NT5E	−1.10	0.745993	0.886751
55872	PBK	−1.54	0.036326	0.10184
8993	PGLYRP1	1.68	0.967955	1
5467	PPARD	−1.02	0.628708	0.824119
5468	PPARG	−2.82	1.00E-08	2.26E-07
5580	PRKCD	−1.52	0.000383	0.00232
5692	PSMB4	−1.64	0.000331	0.002054
5734	PTGER4	1.64	0.000637	0.003575
5740	PTGIS	2.51	0.032953	0.094137
5771	PTPN2	−1.08	0.566503	0.777092
27342	RABGEF1	1.06	0.659667	0.843973
6223	RPS19	−2.16	1.32E-07	2.15E-06
6288	SAA1	1.56	0.332259	0.548654
55829	SELS	−1.44	0.010148	0.036066
462	SERPINC1	−5.45	0.002483	0.011294
5176	SERPINF1	2.70	3.37E-10	1.17E-08
81858	SHARPIN	−1.92	2.10E-06	2.50E-05
4088	SMAD3	−1.65	1.94E-05	0.000176
9021	SOCS3	1.73	0.004227	0.017615

6693	SPN	3.36	3.34E-10	1.17E-08
7032	TFF2	−1.85	0.215818	0.404138
7128	TNFAIP3	2.69	2.90E-09	7.66E-08
79626	TNFAIP8L2	2.12	0.001088	0.005629
7132	TNFRSF1A	−1.11	0.349323	0.565869
7133	TNFRSF1B	1.84	0.000111	0.0008
7301	TYRO3	−1.32	0.167036	0.333937
55075	UACA	1.31	0.091579	0.211729
7538	ZFP36	1.06	0.780563	0.908267

PDAC: Pancreatic Ductal Adenocarcinoma; FDR: False Discovery Rate; 17 genes down-regulated in Strong Angiogenic PDAC vs. Weak Angiogenic PDAC; 33 genes differentially expressed in Strong Angiogenic PDAC vs. Weak Angiogenic PDAC (16 up-regulated, 17 down-regulated); Differential expression cut off: |Fold Change| > = 1.5, FDR < 0.05.

Supplementary Table S5: Expression of 85 positive regulators of inflammation in strong angiogenic PDAC vs. weak angiogenic PDAC

Entrez Gene ID	Gene Symbol	Strong Angiogenic PDAC vs. Weak Angiogenic PDAC		
		Fold Change	*P*-value	FDR
101	ADAM8	−2.18	1.25E-07	2.07E-06
136	ADORA2B	−1.40	0.327524	0.543411
140	ADORA3	2.78	4.72E-08	8.75E-07
185	AGTR1	2.35	0.000345	0.002126
338699	ANKRD42	1.02	0.898732	0.986776
6369	CCL24	−6.02	0.265392	0.468215
6348	CCL3	1.78	0.037072	0.103521
1236	CCR7	8.60	0.004296	0.017835
1E+08	CD24	−1.23	0.345747	0.562815
940	CD28	8.26	3.22E-11	1.50E-09
961	CD47	1.03	0.832139	0.942403
9575	CLOCK	2.16	1.01E-05	9.99E-05
1268	CNR1	5.38	1.65E-10	6.31E-09
84699	CREB3L3	−2.87	0.401734	0.621051
1520	CTSS	1.11	0.57403	0.783145
6376	CX3CL1	−1.13	0.395828	0.615206
1909	EDNRA	2.53	4.40E-09	1.10E-07
1956	EGFR	1.70	0.00155	0.007595
2161	F12	−6.21	3.35E-15	5.68E-13
2167	FABP4	3.31	0.034416	0.097503
2205	FCER1A	2.66	0.000966	0.005082
2207	FCER1G	1.89	0.000282	0.001785
2867	FFAR2	−1.97	0.450042	0.671311
2865	FFAR3	1.32	0.561418	0.773537
51704	GPRC5B	1.04	0.893443	0.983375
8692	HYAL2	1.21	0.194683	0.374217
3620	IDO1	2.81	0.239865	0.435599
3593	IL12B	3.16	0.334213	0.550746
3600	IL15	1.13	0.696756	0.858676
3605	IL17A	9.42	1	1
27190	IL17B	1.30	0.919437	1
27189	IL17C	−4.67	0.044896	0.120851
112744	IL17F	−2.45	0.649887	0.838644

23765	IL17RA	1.29	0.173192	0.342351
84818	IL17RC	−1.77	2.58E-05	0.000226
3606	IL18	−1.24	0.092931	0.214054
9173	IL1RL1	3.88	0.004441	0.01831
3558	IL2	2.88	0.772575	0.902868
59067	IL21	Inf	1	1
51561	IL23A	−1.46	0.107447	0.239118
90865	IL33	5.12	1.03E-12	7.78E-11
3569	IL6	4.06	0.008857	0.032238
3572	IL6ST	5.81	9.39E-20	5.49E-17
3673	ITGA2	1.34	0.099583	0.226062
3717	JAK2	1.59	0.008254	0.030425
3818	KLKB1	1.00	0.974636	1
3929	LBP	2.91	0.19577	0.375759
4049	LTA	4.90	0.001887	0.008958
5603	MAPK13	−1.56	0.006447	0.024891
91662	NLRP12	2.60	0.072132	0.175901
4889	NPY5R	1.24	0.701563	0.861658
5008	OSM	1.37	0.371834	0.589543
9180	OSMR	2.27	4.31E-07	6.15E-06
5138	PDE2A	2.81	7.58E-10	2.44E-08
5320	PLA2G2A	1.30	1	1
7941	PLA2G7	2.47	0.021131	0.065952
5733	PTGER3	3.79	3.96E-13	3.43E-11
5734	PTGER4	1.64	0.000637	0.003575
5743	PTGS2	1.47	0.55703	0.770377
6223	RPS19	−2.16	1.32E-07	2.15E-06
6283	S100A12	3.55	0.083594	0.196844
6279	S100A8	−1.00	0.698008	0.85942
6280	S100A9	−1.02	0.764485	0.898862
5054	SERPINE1	3.65	7.38E-07	9.88E-06
8723	SNX4	1.06	0.726897	0.875185
6776	STAT5A	1.13	0.59753	0.801278
6777	STAT5B	1.42	0.022094	0.06837
6863	TAC1	−1.45	0.383751	0.603045
7052	TGM2	1.48	0.099061	0.225149
81793	TLR10	9.80	5.76E-05	0.000455
7097	TLR2	1.83	0.000326	0.002029
7098	TLR3	1.37	0.107796	0.239626
7099	TLR4	3.10	3.33E-11	1.55E-09

51284	TLR7	4.61	1.05E-11	5.77E-10
54106	TLR9	1.93	0.056105	0.14437
7124	TNF	−1.04	0.568062	0.778033
8792	TNFRSF11A	−1.41	0.168751	0.336384
7132	TNFRSF1A	−1.11	0.349323	0.565869
8600	TNFSF11	−1.59	0.093752	0.215598
7292	TNFSF4	2.06	0.002548	0.011533
10318	TNIP1	−1.28	0.055041	0.142091
6845	VAMP7	1.65	0.000551	0.003168
8673	VAMP8	−2.01	3.11E-07	4.58E-06
7474	WNT5A	1.77	0.00685	0.026145
7784	ZP3	−2.28	1.80E-05	0.000165

PDAC: Pancreatic Ductal Adenocarcinoma; FDR: False Discovery Rate; 28 genes up-regulated in Strong Angiogenic PDAC vs. Weak Angiogenic PDAC; 35 genes differentially expressed in Strong Angiogenic PDAC vs. Weak Angiogenic PDAC (28 up-regulated, 7 down-regulated); Differential expression cut off: |Fold Change| > = 1.5, FDR < 0.05.

Supplementary Table S6: Expression of 45 JAK/STAT signaling pathway genes in strong and moderate angiogenic PDAC vs. weak angiogenic PDAC

Entrez Gene ID	Gene Symbol	Strong Angiogenic PDAC vs. Weak Angiogenic PDAC			Moderate vs. Weak Angiogenic PDAC		
		Fold Change	*P*-value	FDR	Fold Change	*P*-value	FDR
10000	AKT3	3.42	7.12E-15	1.05E-12	2.09	2.91E-07	8.88E-05
867	CBL	1.66	0.003123	0.013644	1.32	0.123839	0.451204
23624	CBLC	−4.26	7.19E-25	2.04E-21	−1.68	6.96E-05	0.004001
1271	CNTFR	1.56	0.664617	0.847142	−4.02	0.001979	0.037008
1438	CSF2RA	2.36	1.49E-05	0.00014	1.28	0.258325	0.660969
1439	CSF2RB	4.98	2.82E-07	4.21E-06	1.96	0.025106	0.181024
1441	CSF3R	2.68	0.000418	0.002506	1.73	0.056468	0.29521
2033	EP300	1.64	0.00059	0.003353	1.32	0.049323	0.272434
2057	EPOR	−1.51	0.011135	0.038858	−1.16	0.264323	0.669341
2690	GHR	4.99	8.99E-06	9.01E-05	1.75	0.047669	0.267402
338376	IFNE	−7.53	0.002234	0.010338	−1.85	0.279327	0.6884
3459	IFNGR1	1.56	0.002345	0.010776	1.26	0.098858	0.399409
3587	IL10RA	3.23	7.52E-11	3.12E-09	1.33	0.119316	0.442423
3594	IL12RB1	1.91	0.014321	0.047841	1.09	0.695597	0.934695
50615	IL21R	3.43	1.10E-05	0.000108	1.66	0.053024	0.285062
3559	IL2RA	3.84	3.93E-08	7.47E-07	1.88	0.012143	0.115874
3560	IL2RB	3.42	4.88E-09	1.19E-07	1.54	0.021141	0.163487
3561	IL2RG	−1.73	0.004305	0.017862	−1.48	0.048387	0.269966
3563	IL3RA	1.58	0.007286	0.027509	1.09	0.525521	0.891302
3569	IL6	4.06	0.008857	0.032238	1.01	0.817692	0.983347
3570	IL6R	2.86	6.32E-05	0.000494	1.41	0.163812	0.526778
3572	IL6ST	5.81	9.39E-20	5.49E-17	2.43	3.26E-07	9.02E-05
3575	IL7R	9.98	9.41E-18	3.66E-15	2.54	0.000195	0.008046
3716	JAK1	1.72	0.000154	0.001067	1.32	0.038427	0.233472
3717	JAK2	1.59	0.008254	0.030425	−1.03	0.649789	0.918528
3718	JAK3	2.25	0.000455	0.002697	1.25	0.353542	0.764938
3952	LEP	7.28	0.014287	0.047744	−2.01	0.381413	0.78991
3953	LEPR	3.87	1.75E-09	4.95E-08	1.86	0.001661	0.033534
3977	LIFR	2.30	2.48E-06	2.87E-05	−1.09	0.715637	0.943575
9180	OSMR	2.27	4.31E-07	6.15E-06	1.86	9.51E-05	0.005001
9063	PIAS2	1.95	0.000441	0.002626	1.23	0.23986	0.638306
5290	PIK3CA	2.24	1.89E-06	2.27E-05	1.65	0.002061	0.037797
5293	PIK3CD	2.81	1.21E-08	2.66E-07	1.60	0.00454	0.062265

5294	PIK3CG	5.16	1.46E-12	1.06E-10	1.96	0.0031	0.049477
5295	PIK3R1	2.41	1.71E-09	4.83E-08	1.41	0.006457	0.076798
8503	PIK3R3	1.79	0.000155	0.001073	1.16	0.293415	0.705203
23533	PIK3R5	3.91	7.50E-13	5.93E-11	1.68	0.003025	0.048665
8835	SOCS2	1.90	5.22E-05	0.000418	−1.03	0.863102	1
9021	SOCS3	1.73	0.004227	0.017615	1.13	0.607341	0.909456
9655	SOCS5	1.82	0.00014	0.00098	1.39	0.02991	0.202197
30837	SOCS7	1.91	0.011688	0.040439	1.39	0.132412	0.467962
6655	SOS2	1.58	0.001599	0.007794	1.29	0.053097	0.285225
10252	SPRY1	2.23	4.05E-08	7.65E-07	1.30	0.040648	0.242245
6772	STAT1	1.56	0.012906	0.043866	1.36	0.068442	0.327722
6775	STAT4	3.22	1.17E-07	1.96E-06	1.21	0.233588	0.631481

PDAC: Pancreatic Ductal Adenocarcinoma; FDR: False Discovery Rate; 44 differentially expressed genes in Strong Angiogenic PDAC vs. Weak Angiogenic PDAC (40 up-regulated, 4 down-regulated); 10 differentially expressed genes in Moderate Angiogenic PDAC vs. Weak Angiogenic PDAC (8 up-regulated, 2 down-regulated); Differential expression cut off: |Fold Change| > = 1.5, FDR < 0.05.
